# Functional characterisation of the transcriptome from leaf tissue of the fluoroacetate-producing plant, *Dichapetalum cymosum*, in response to mechanical wounding

**DOI:** 10.1038/s41598-020-77598-7

**Published:** 2020-11-25

**Authors:** Selisha A. Sooklal, Phelelani T. Mpangase, Mihai-Silviu Tomescu, Shaun Aron, Scott Hazelhurst, Robert H. Archer, Karl Rumbold

**Affiliations:** 1 School of Molecular and Cell Biology, University of the Witwatersrand, Johannesburg, 2000 South Africa; 2 Sydney Brenner Institute for Molecular Biosciences, University of the Witwatersrand, Johannesburg, 2000 South Africa; 3 National Herbarium, South African National Biodiversity Institute, Pretoria, 0186 South Africa

**Keywords:** Wounding, Gene expression profiling, Transcriptomics, Transferases, Bioinformatics

## Abstract

*Dichapetalum cymosum* produces the toxic fluorinated metabolite, fluoroacetate, presumably as a defence mechanism. Given the rarity of fluorinated metabolites in nature, the biosynthetic origin and function of fluoroacetate have been of particular interest. However, the mechanism for fluorination in *D. cymosum* was never elucidated. More importantly, there is a severe lack in knowledge on a genetic level for fluorometabolite-producing plants, impeding research on the subject. Here, we report on the first transcriptome for *D. cymosum* and investigate the wound response for insights into fluorometabolite production. Mechanical wounding studies were performed and libraries of the unwounded (control) and wounded (30 and 60 min post wounding) plant were sequenced using the Illumina HiSeq platform. A combined reference assembly generated 77,845 transcripts. Using the SwissProt, TrEMBL, GO, eggNOG, KEGG, Pfam, EC and PlantTFDB databases, a 69% annotation rate was achieved. Differential expression analysis revealed the regulation of 364 genes in response to wounding. The wound responses in *D. cymosum* included key mechanisms relating to signalling cascades, phytohormone regulation, transcription factors and defence-related secondary metabolites. However, the role of fluoroacetate in inducible wound responses remains unclear. Bacterial fluorinases were searched against the *D. cymosum* transcriptome but transcripts with homology were not detected suggesting the presence of a potentially different fluorinating enzyme in plants. Nevertheless, the transcriptome produced in this study significantly increases genetic resources available for *D. cymosum* and will assist with future research into fluorometabolite-producing plants.

## Introduction

Fluorinated metabolites are extremely rare in nature relative to other halogenated natural products^1,2^. This is largely a consequence of the low bioavailability of fluorine, the high energy cost associated with desolvating the fluoride ion and exclusion from enzymatic incorporation via halogenases which utilise oxidative catalytic mechanisms^2,3^. Despite these factors, a fluorinated secondary metabolite was isolated from the plant *Dichapetalum cymosum*^4,5^.

*D. cymosum*, or more commonly known as gifblaar (Afrikaans for poison leaf), is a member of the *Dichapetalaceae* family characterised by its shrub-like appearance and extensive underground stem system^6^. This highly toxic plant is scattered in South Africa, Namibia, Zimbabwe, Botswana and Angola^7^. After observing sudden death in animals that ingested *D. cymosum*, Marais isolated fluoroacetate as the constituent responsible for toxicity^4,5^. This marked the discovery of the first fluorinated metabolite from a biological source. *D. cymosum* was subsequently shown to accumulate fluoroacetate at concentrations up to 232 mg kg^−1^ in the young leaves, 97 mg∙kg^−1^ in the older leaves, 164 mg∙kg^−1^ in the immature seeds and 362 mg∙kg^−1^ in the flowers^8^. Toxicity is effectuated through ‘lethal synthesis’ whereby fluoroacetate is metabolised to (2*R*, 3*R*)-2-fluorocitrate in vivo. Coincidently, this is the only stereoisomer of fluorocitrate that is an inhibitor of aconitate hydratase (a key enzyme in the tricarboxylic acid cycle) thus terminating cellular respiration^9,10^. The presence of fluoroacetate has been confirmed in just over 30 plants worldwide, including 8 species from the *Dichapetalaceae* family^11^.

The production of fluoroacetate is widely believed to be a defence mechanism in plants, however this hypothesis was never investigated experimentally^12^. Instead, given how rare fluorometabolites are in nature, there was far more interest in the mechanisms underlying the biosynthesis of this fluorinated metabolite. Explanations such as the acquisition of fluoroacetate from microorganisms in the soil surrounding *D. cymosum* and fluoroacetate-producing endosymbionts have been proposed^13,14^. However, later work by Grobbelaar and Meyer showed that a callus of *D. cymosum*, devoid of contaminating microorganisms, had the ability to produce fluoroacetate when supplemented with fluoride ions^15^. These findings suggested the evolution of an enzyme with the capacity to incorporate fluorine into organic molecules. However, further attempts to clarify the mechanism for fluorination in *D. cymosum* were challenged by access to plant material and a general lack of knowledge regarding the natural substrate for such an enzyme^15–17^. Therefore, little progress was achieved and the production of fluoroacetate remains a mystery in higher plants.

Attention quickly shifted to another fluorometabolite-producer, *Streptomyces cattleya*. This bacterium, which produces fluoroacetate as well as 4-fluorothreonine, provided a practical biological system to study fluorination^18^. Extensive efforts led to the discovery of the enzyme 5′-fluoro-5′-deoxyadenosine synthase (fluorinase) and elucidation of the fluorometabolite biosynthetic pathway in prokaryotes^2,19,20^. Fluorinases mediate the conversion of *S*-adenosyl-L-methionine (SAM) and inorganic fluoride ions into 5′-fluoro-5′-deoxyadenosine (5′-FDA) and L-methionine. This constitutes the first step in fluorometabolite biosynthesis. The pathway then proceeds with five additional enzymatic steps to yield fluoroacetate or 4-fluorothreonine. With the exclusive ability to catalyse the formation of a C–F bond, it is no surprise that fluorinases have significant potential in biotechnological applications^2^.

Previous studies into *D. cymosum* were largely conducted on a physiological level. The progression of modern technologies such as next-generation sequencing (NGS) has allowed for the efficient profiling of non-model organisms, like *D. cymosum*, and has become the standard tool for simultaneous gene discovery and RNA quantification^21^. However, there remains a severe lack in genetic resources available for this plant with only one sequence *rbcLa* (KF147466.1) on the GenBank database. Moreover, wound responses such as the elicitation of signalling cascades, regulation of phytohormones and liberation of secondary metabolites are well characterised in model species like *Arabidopsis thaliana*; but there are no prior reports detailing the wound response in *D. cymosum*^22,23^.

Herein, we explored the transcriptome of *D. cymosum* in response to mechanical wounding. Libraries were generated prior to mechanical wounding (T:0), as well as 30 min (T:30) and 60 min (T:60) post wounding. To the best of our knowledge, this is the first transcriptome of *D. cymosum*. Therefore, a combined reference transcriptome was created and comprehensively characterised, significantly expanding genetic resources available for this plant. In addition, the regulation of key processes in response to wounding was investigated, providing insights into the molecular mechanisms governing inducible defences in *D. cymosum*.

## Results

### Sequencing and de novo assembly of the *D. cymosum* transcriptome

In this study, *D. cymosum* leaves were challenged with mechanical wounding. cDNA libraries of replicates obtained from the control (T:0) and time points post wounding (T:30 and T:60) were sequenced using the Illumina HiSeq 2500 platform. At present, there is virtually no sequence information available for this plant with the GenBank database containing only 1 entry. Therefore, to develop the first overview of the *D. cymosum* transcriptome, the data from the three libraries and their respective replicates were pooled to produce 60,638,658 raw paired-end (PE) reads with an average length of 125 bp. Following the data filtering process, an enhancement in the average quality (≥ 30 Phred score) at each base position of the sequence reads was seen (Supplementary Fig. S1) and a total of 52,819,612 (87%) high-quality PE reads with a sequence length of 50–125 bp remained. The reads were then assembled de novo using Trinity. Trinity produced 77,845 transcripts with an average length and N50 of 454 bp and 480 bp, respectively. The shortest transcript generated was 224 bp while the longest transcript had a length of 10,795 bp. The length distribution of transcripts is depicted in Supplementary Fig. S2. In addition, a GC content of 41.8% was observed. The completeness of the assembled transcriptome was assessed using BUSCO (eudicotyledons). The *D. cymosum* transcriptome contained 17.1% complete genes (13.1% were single-copy and 4% were duplicated) and 26.3% fragmented genes. In total, 43.4% of the benchmarked orthologs used by BUSCO were accounted for in this transcriptome while 56.6% were denoted missing. A summary of the sequencing, data filtering, assembly and transcriptome completeness outputs are shown in Table 1.

**Table 1:** Summary of the sequencing, data filtering, assembly and transcriptome completeness output for the *D. cymosum* transcriptome.

**Sequencing output**	
Number of raw reads	60,638,658
Read length (bp)	125
**Quality filtering output**	
Number of high-quality reads	52,819,612
Read length (bp)	50–125
Number of PE reads used in assembly	26,409,806
**Assembly output**	
Total Trinity genes	64,325
Total Trinity transcripts	77,845
Average contig length (bp)	454
N50 (bp)	480
Percent GC	41.8
**Transcriptome completeness output**	
BUSCO eudicotyledons	C: 17.1% [S: 13.1%, D: 4.0%]
	F: 26.3%
	M: 56.6%

### Functional characterisation of the *D. cymosum* transcriptome

#### Annotation overview

Sequencing of plant transcriptomes typically results in large amounts of deduced gene or protein sequences, of which, very few will ever be investigated experimentally. Annotation through comparative sequence analysis provides the only feasible route to assign putative functions^21^. Therefore, sequence similarity searches were conducted against various databases (SwissProt, TrEMBL, GO, eggNOG/COG, KEGG, Pfam, ExPASy-Enzyme and PlantTFDB). Collectively, a 69% annotation rate was achieved for the *D. cymosum* transcriptome using the aforementioned databases (Table 2) with majority of the transcripts being identified by TrEMBL. The organisms from which annotations were derived, was subsequently explored. The species distribution (Supplementary Fig. S3) showed that most sequences exhibited homology to *Arabidopsis thaliana* (27%). This was followed by *Populus trichocarpa*, (12.3%), *Jatropha curcas* (10.6%), and *Ricinus communis* (10.5%).

**Table 2:** Summary of the annotation output for the *D. cymosum* transcriptome.

Database	Number of annotated transcripts
SwissProt	36,053
TrEMBL	53,452
GO	42,664
eggNOG	19,085
COG	18,690
KEGG	10,138 transcripts, 125 pathways

Database	Number of annotated proteins
Pfam	23,878
ExPASy-Enzyme	7311
PlantTFDB	1220

#### GO and eggNOG

Overall, 42,664 (58%) transcripts were assigned GO terms. In many cases, multiple GO terms were assigned to the same transcript thus resulting in the molecular function, cellular component and biological process domains containing 35,709; 31,638 and 31,136 transcripts, respectively. Using a set of plant-specific GO Slims, annotations were further sub-divided into 97 functional terms (Supplementary Fig. S4). Amongst the molecular function category; ‘nucleotide binding’ and ‘binding’ were the most represented groups. Within the cellular component category, a high percentage of transcripts were assigned to ‘membrane’. For biological process, ‘cellular process’ and ‘metabolic process’ were the most dominant groups. Interestingly, it was observed that ‘response to stress’ and ‘signal transduction’ were also among the top represented groups in biological process. Since majority of the reads in this transcriptome were from the wounded *D. cymosum* leaves (T:30 + T:60), it is not atypical to see a number of transcripts directed towards stress and recovery responses of the plant.

**Figure 1:**
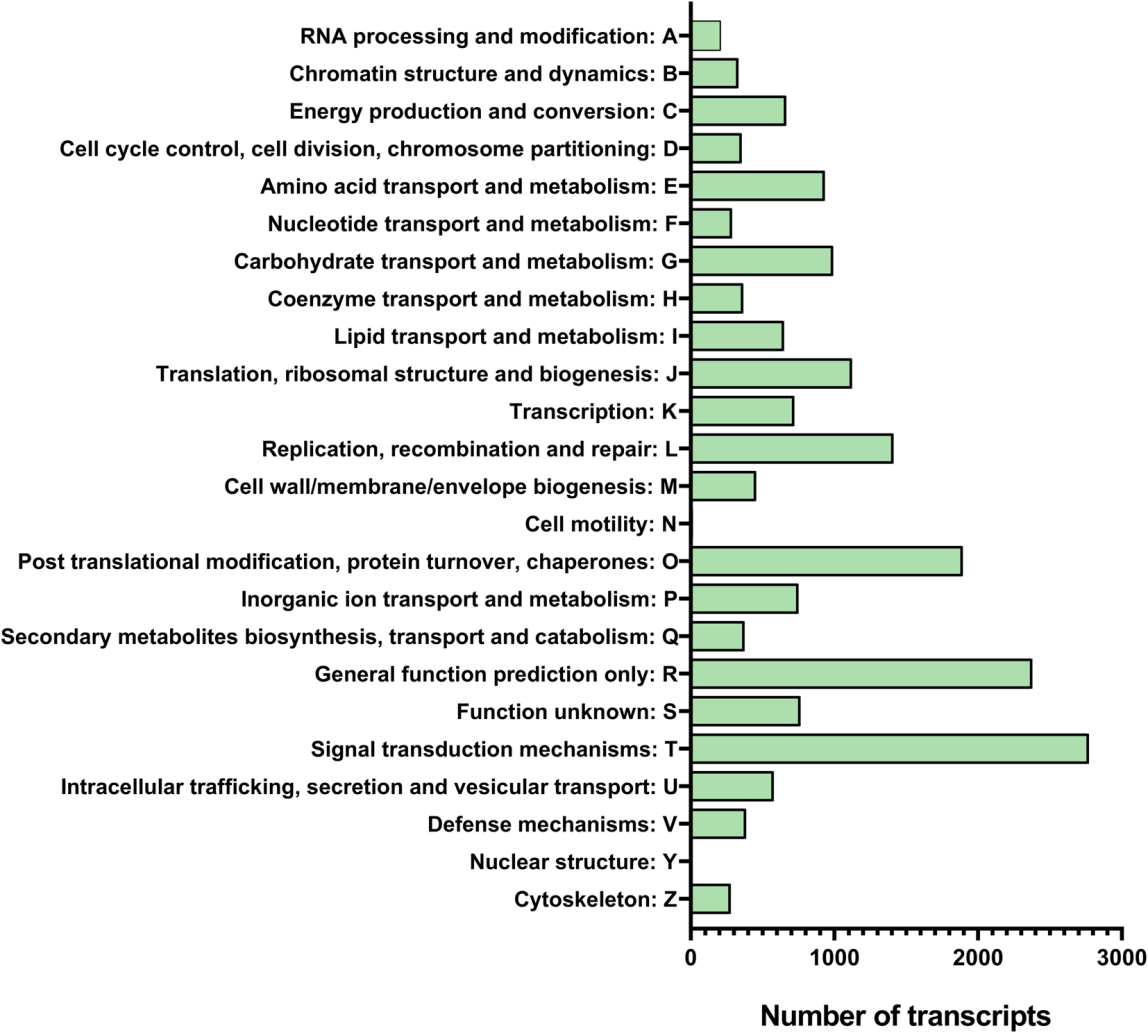
COG classification of *D. cymosum* transcripts. A total of 19,085 transcripts were identified by the eggNOG database. Of these, 18,690 were categorised into 25 COG clusters.

Orthologous inferences were made using eggNOG. Here, 19,085 transcripts were identified, of which, 18,690 received single-letter codes as defined for COG to allow functional clustering into fewer groups (Fig. 1). ‘Signal transduction mechanisms’ was the most abundant group, again, suggesting the common response of a wounded plant. This was followed by ‘general function prediction only’.

#### KEGG

**Figure 2:**
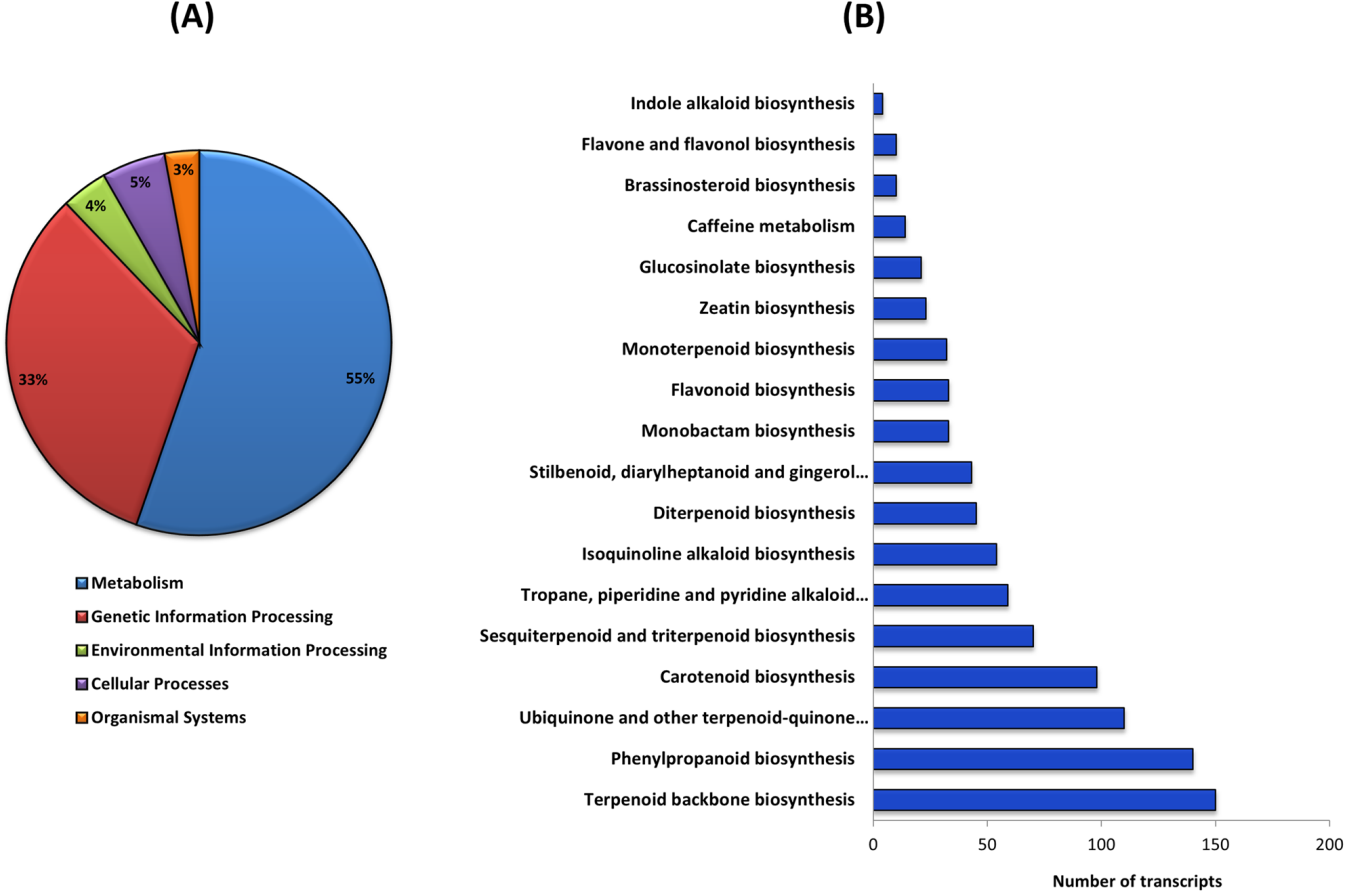
(**A**) Distribution of 10,138 transcripts into the broader metabolism, genetic information, environmental information processing, cellular processes and organismal systems categories in KEGG. (**B**) Allocation of transcripts within ‘metabolism’ into selected secondary metabolic pathways.

Active pathways in the *D. cymosum* transcriptome were identified using *A. thaliana* as a reference on the KEGG database. KEGG successfully mapped 10,138 individual transcripts to 125 unique pathways within the ‘metabolism’, ‘genetic information processing’, ‘environmental information processing’, ‘cellular processes’ and ‘organismal systems’ categories (Fig. 2A). Pathways within the ‘metabolism’ category accounted for 55% of the aforementioned transcripts. Of these transcripts, nearly 10% were allocated to selected secondary metabolic processes, as shown in Fig. 2B. The ‘terpenoid backbone biosynthesis’, phenylpropanoid biosynthesis’ and ‘ubiquinone and other terpenoid-quinone biosynthesis’ secondary metabolic pathways were highly represented.

#### SSR’s

In total, 1534 Simple Sequence Repeats (SSR’s) were discovered in 1442 *D. cymosum* transcript sequences. Of these, 91 sequences contained more than one SSR. The trinucleotide motifs accounted for 57.8% (887) of all SSR’s. This was followed by dinucleotide motifs with 25.6% (393) of SSR’s (Supplementary Table S1). The most abundant repeat sequence for the trinucleotide motifs were CTT/AAG and AG/CT for the dinucleotide motifs. In addition, 51 SSR’s were detected in a complex formation.

#### Pfam and EC

Overall, 36,528 ORFs of at least 100 amino acids long were identified. The predicted protein sequences were queried against the Pfam database to extract conserved domain information. Of these, 23,878 (65.4%) sequences contained at least one Pfam domain. Collectively, 8496 different Pfam domains were ultimately assigned with the ‘protein kinase’ and ‘protein tyrosine kinase’ domains being the most predominant by far. In addition, 976 unique EC numbers were retrieved from ExPasy for 7311 protein sequences. EC numerically classified enzymes into 6 categories based on catalytic function (Supplementary Fig. S5). Specifically, these were transferases (45%), hydrolases (27%), oxidoreductases (14%), lyases (5%), ligases (5%) and isomerases (4%).

#### Transcription factors

**Figure 3:**
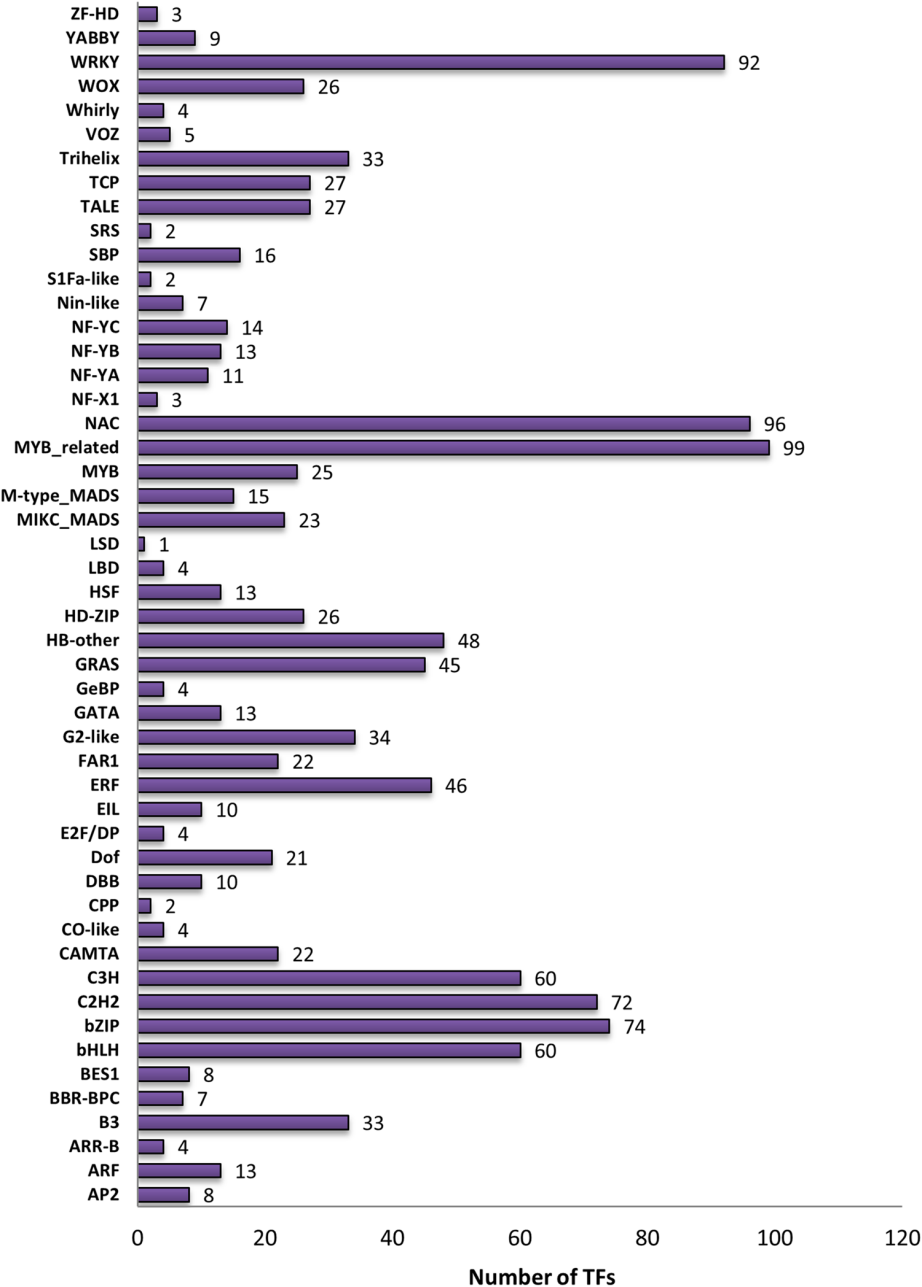
The families of transcription factors detected in the *D. cymosum* transcriptome. A total of 1220 transcription factors were assigned to 50 unique families using PlantTFDB.

In all, 1220 transcription factors from 50 unique families were found in the *D. cymosum* transcriptome. The most number of transcription factors (99) were from the MYB-related family. This was followed closely by the NAC and WRKY families containing 96 and 92 transcription factors, respectively (Fig. 3).

### Investigating the *D. cymosum* transcriptome for fluorometabolite biosynthetic genes

Following extensive annotation of the *D. cymosum* transcriptome, only four putative fluoride ion transporters were detected (TR45147_c0_g1_i1, TR45147_c0_g1_i2, TR45147_c0_g1_i3 and TR45147_c0_g1_i4) and transcripts associated with fluorinase activity were not found. Consequently, the enzymes involved in the *S. cattleya* fluorometabolite biosynthesis pathway were queried against the *D. cymosum* transcriptome in order to identify any potential hits that may have been annotated fallaciously or not annotated at all. The best hit for each enzyme is summarised in Supplementary Table S2. Hits in the *D. cymosum* transcriptome exhibited low identity to the enzymes from *S. cattleya*. Moreover, the matches had high e-values, low bit scores and were further complicated by short alignment lengths suggesting that no significant match was found, except for an aldolase (56% identity) and an aldehyde dehydrogenase (41.5% identity).

### Characterisation of the wound response in *D. cymosum*

**Figure 4:**
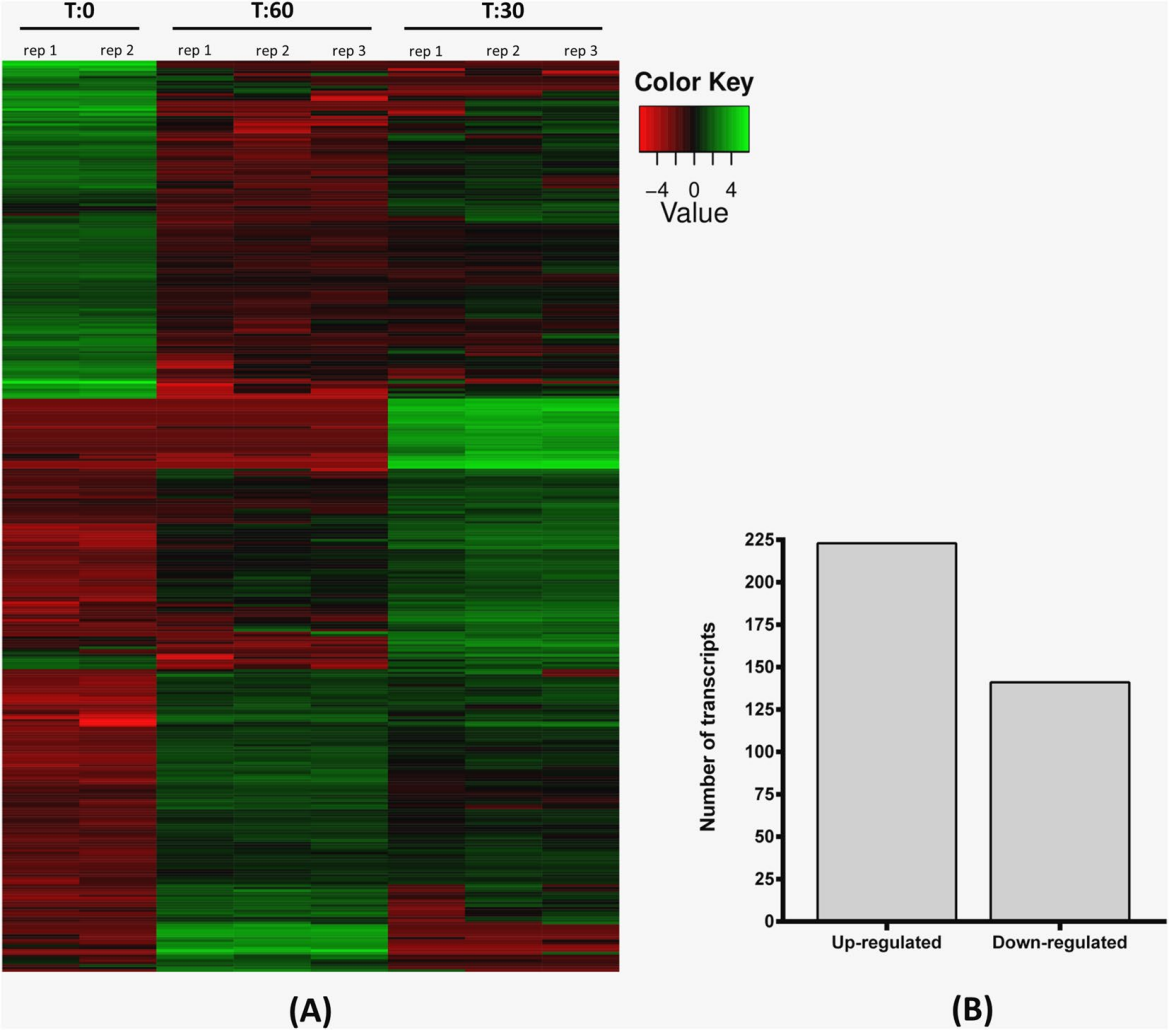
(**A**) Clustered heatmap showing the differential regulation (FDR ≤ 0.001) of 364 genes in response to mechanical wounding in *D. cymosum*. Green indicates up-regulation while red indicates down-regulation. (**B**) Of the 364 DEGs detected, 223 were up-regulated and 141 were down-regulated.

Until now, there have been no reports detailing the metabolic adjustments of *D. cymosum* in response to wounding. Hence the genetic changes in *D. cymosum* at two stages following mechanical wounding were characterised by aligning the reads from each time point (T:0, T:30 and T:60), and their respective replicates, to the reference transcriptome generated in this study. The correlation matrix showed minor variation in expression between replicates within the same time point indicating reproducibility of the experimental data (Fig. S6). However, as expected, more variation was seen across time points between the healthy and wounded *D. cymosum* leaves. The abundance of each transcript was then estimated and differential expression analysis was conducted using edgeR. Search parameters (FDR ≤ 0.001 and ≥ fourfold change between two time points) limited DEGs to only those that were the most significantly differentially regulated and, hence, are pivotal in the wound response. Using this approach, 364 DEGs were identified (Fig. 4A). Of these, 223 DEGs were transiently up-regulated in at least one time point post wounding and, conversely, 141 DEGs were transiently down-regulated in at least one time point post wounding (Fig. 4B).

#### Enriched gene ontologies of DEGs

**Figure 5:**
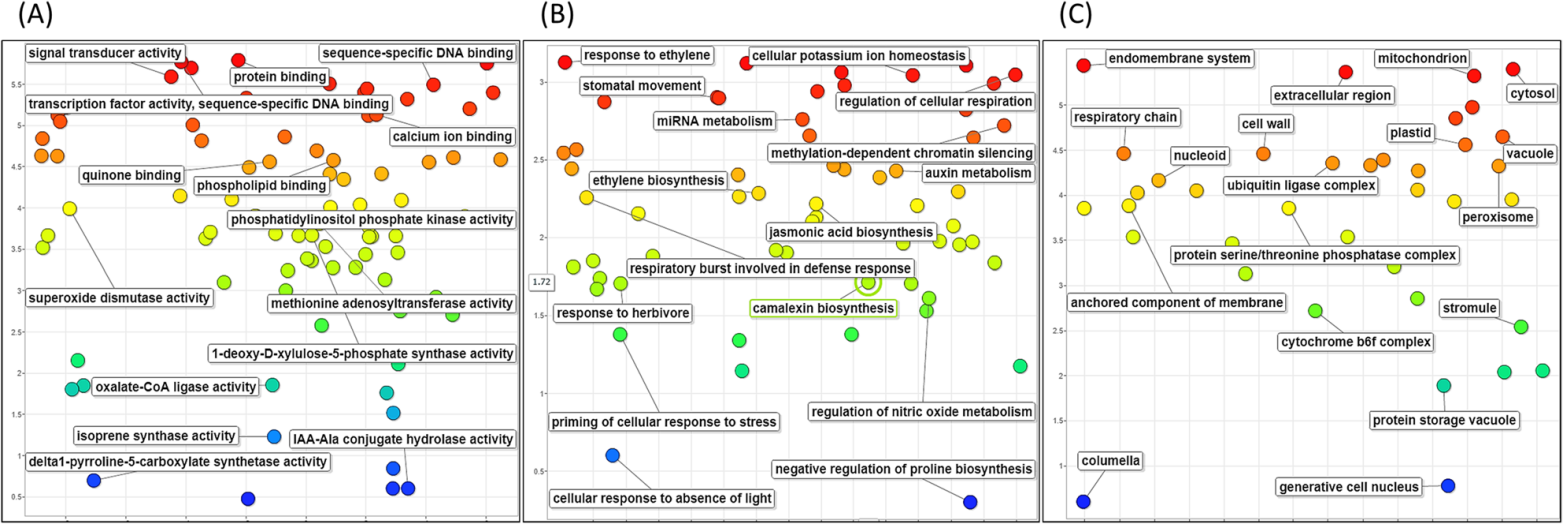
Scatter plot of the GO enrichment for DEGs in response to mechanical wounding in *D. cymosum*. Enriched DEGs were classified into the (**A**) molecular function (**B**) biological process and (**C**) cellular component categories. The plot is based on the frequency of the GO terms rather than representing the actual regulation of each gene. The log count is shown on the y-axis. Semantic space is represented on the x-axis.

In total, 278 DEGs were annotated with 1972 GO terms. The molecular function, cellular component and biological process categories contained 236, 213 and 247 transcripts, respectively. Enrichment of GO terms was then carried out using REVIGO. Enrichment allowed for functional profiling of DEGs by finding a subset of representative terms in order to elucidate underlying mechanisms associated with the wound response in *D. cymosum*. These processes are shown on a scatter plot (Fig. 5). The log count of GO terms for each subcategory is represented on the y-axis as well as by the colour of the bubble. The x-axis is ordered based on semantic differences between GO terms. For molecular function, it is evident that ‘signal transducer activity’, ‘transcription factor activity’ and ‘calcium ion binding’ are among the most enriched processes. For biological process, ‘response to ethylene’ was most represented. Of note were also ‘ethylene biosynthesis’, ‘jasmonic acid biosynthesis’ and ‘camalexin biosynthesis’. For the cellular component category, the ‘endomembrane system’, ‘mitochondrion’ and ‘cell wall’ were prioritised. It is important to note, however, that the plot is based on the frequency of the GO terms rather than representing the actual regulation of each gene. This means that if a subcategory appears lower down on the plot, there are fewer terms associated with this category rather than the process being down-regulated.

#### Regulation of DEGs

**Figure 6:**
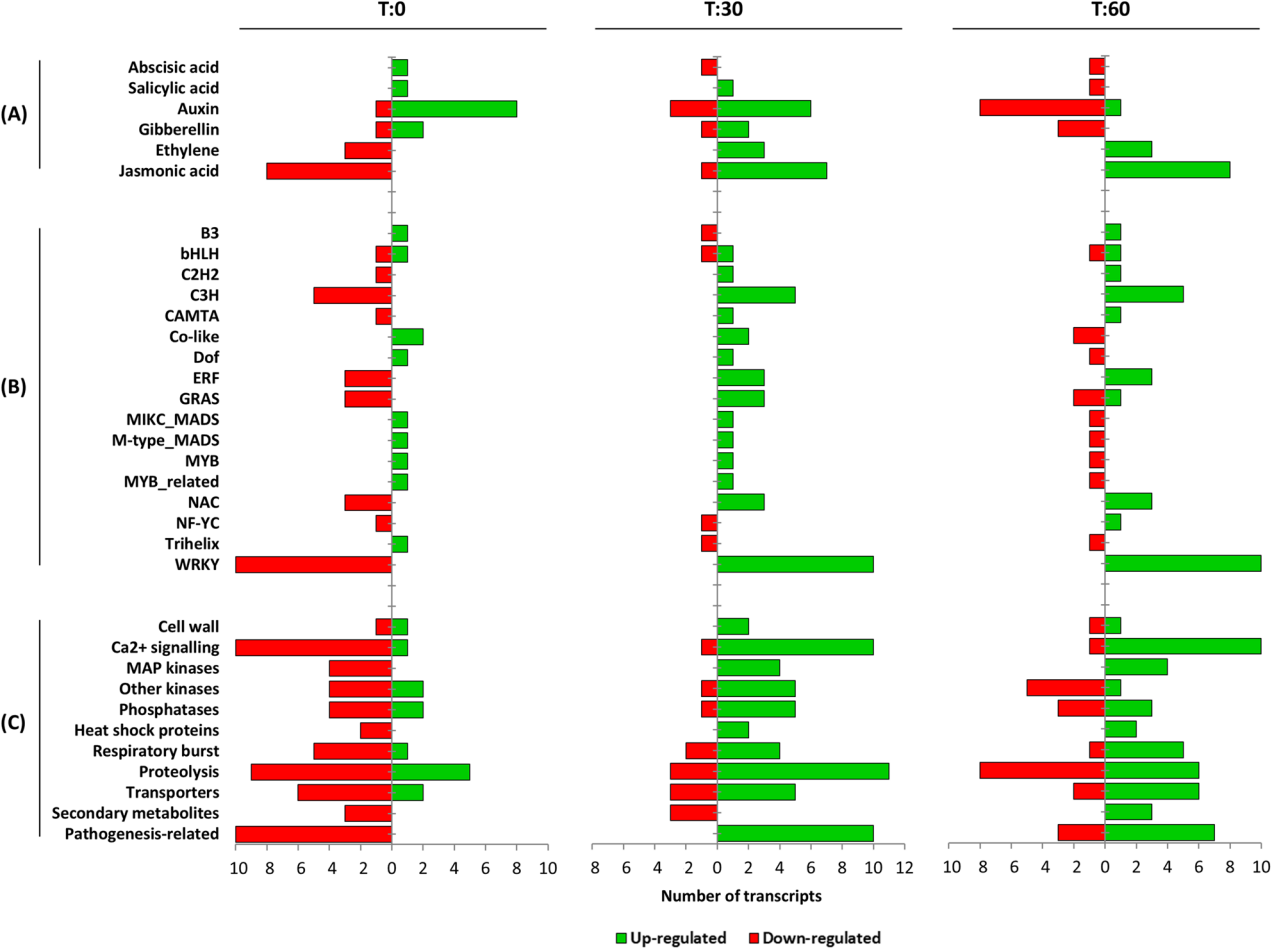
The number of DEGs either up-regulated (green) or down-regulated (red) in wound-responsive processes relating to (**A**) phytohormones, (**B**) transcription factors and (**C**) other significant processes in *D. cymosum*.

In comparison to the enriched GO plots, the absolute regulation of each gene was also investigated. For this, annotations for 247 DEGs were retrieved from the SwissProt database and transcripts were arranged based on putative function. Various *D. cymosum* genes associated with phytohormones, transcription factors, signal transduction mechanisms, redox regulation, proteolysis, secondary metabolite biosynthesis and pathogenesis-responsive pathways were found to be differentially regulated in response to mechanical wounding. Figure 6 and Supplementary Table S3 shows the number of DEGs associated with these responses that were either up-regulated or down-regulated over the time course post wounding.

The analysis of phytohormones (Fig. 6A, Supplementary Table S3) indicated a prominent up-regulation of the jasmonic acid (JA), ethylene (ET) and salicylic acid (SA) pathways following wounding, whereas the abscisic acid (ABA), auxin (IAA) and gibberellin (GA) pathways appeared to be down-regulated in *D. cymosum*. For the JA pathway, this included the persistent up-regulation of genes involved in JA biosynthesis such as linoleate 13S-lipoxygenase (*13-LOX*) and 12-oxophytodienoate reductase 3 (*OPR3*) at T:30 and T:60. In addition, several JASMONATE ZIM-DOMAIN (*JAZ*) homologues were up-regulated too. Genes such as *S-*adenosylmethionine synthase 2 (*SAM2*) and 1-aminocyclopropane-1-carboxylate oxidase (*ACO*) involved in the biosynthesis of ET were rapidly increased (T:30 + T:60) in response to wounding. Consistently, ethylene-responsive transcription factors (ERFs) followed the same regulation. The enhanced disease resistance 2 gene (*EDR2*), a negative regulator of SA-mediated resistance^24^, was expressed at both T:0 and T:30. However, a down-regulation at T:60 was seen suggesting SA activity at a later stage post wounding.

Conversely, the AUX pathway was down-regulated following mechanical wounding. A number of transcripts corresponding to tryptophan aminotransferase-related proteins (*TAR1*), involved in AUX biosynthesis, as well as auxin-responsive proteins decreased in expression levels by T:60. Reduction in expression of the abscisic stress-ripening protein 3 as well as of the gibberellin-2-beta-dioxygenase and gibberellin receptor GID1 by T:60 also indicated the down-regulation of the ABA and GA pathways, respectively.

The regulation of transcription factors constituted the largest group of DEGs. A total of 38 putative transcription factors were detected within 17 families (Fig. 6B, Supplementary Table S3). Notably, transcription factors from the WRKY family (WRKY6, WRKY15, WRKY18, WRKY33, WRKY46 and WRKY53) were most prominently up-regulated at both T:30 and T:60. This was followed by the C3H, ERF and NAC families with consistent up-regulation following wounding. Interestingly, transcription factor families such as the Co-like, MYB, MYB-related and trihelix were systematically down-regulated.

Figure 6C and Supplementary Table S3 presents other processes vital in the wound response of *D. cymosum*. Signal transduction mechanisms included the Ca^2+^ signalling pathway, the production of reactive oxygen species (ROS) and phosphorylation-dephosphorylation events carried out by mitogen-activated protein kinases (MAPK), other kinases and phosphatases. Within the Ca^2+^ signalling pathway, several calcium-transporting ATPases, calmodulins (*CaM1*), calmodulin-like proteins (*CML27* and *CML45*) and calmodulin-binding transcription activators were rapidly up-regulated at both T:30 and T:60. In stark contrast, a calcineurin B-like protein 4 (*CBL4*) was down-regulated. The up-regulation (T:30 and T:60) of a catalase, reticuline oxidase, thioredoxin and glutaredoxin suggested a respiratory burst following wounding. Expression levels of all MAP kinases (including MAPKKK1 and MAPK3) were increased at T:30 as well as T:60, however, other kinases exhibited a mixed regulation with four up-regulated and two down-regulated following wounding. Similarly, of the six protein phosphatases detected, four were up-regulated and two were down-regulated upon wounding. Several disease resistance proteins consistent with pathogenesis-related proteins were also found up-regulated from T:30. Furthermore, the production of heat shock proteins and selected secondary metabolites were induced at a later stage following wounding. Both 1-deoxyxylulose-5-phosphate synthase (*DXPS*) and isoprene synthases (*ISPS*), involved in terpenoid biosynthesis, were up-regulated at T:60 only.

## Discussion

*D. cymosum* is the first plant in which fluoroacetate, a fluorinated organic metabolite, was observed^5^. Given the rarity of the C-F bond in nature, *D. cymosum* represents a potentially significant source for fluorinating enzymes. However, tangible research into *D. cymosum* is outdated and prior attempts to elucidate the fluorination mechanism have been unsuccessful. Genetic information offers a practical alternative to study biological mechanisms in plants^25,26^. But a significant limitation has been the lack of sequence data publically available for *D. cymosum*. In this study, RNA-seq was used to produce the first overview of the transcriptome for *D. cymosum*, as well as to increase our understanding of the wound-response in this plant at two stages following mechanical wounding.

In the absence of previous reports detailing changes in *D. cymosum* post wounding, we opted to analyse early responses (up to 1 h). Sequencing was performed using the Illumina platform and reads obtained from the unwounded (T:0) and wounded (T:30 and T:60) leaves were combined in order to generate a transcriptome representing a broad diversity of genes for *D. cymosum*. This assembly produced 77,845 transcripts. An assessment of the transcriptome completeness indicated that 56.6% of benchmarked orthologues used by BUSCO were missing. Considering that the transcriptome produced in this study originated from leaf tissue only, an incomplete representation can be expected due to tissue specificities. Nevertheless, with the primary goal of identifying a potential fluorinase gene, the assembled transcriptome was extensively characterised using various databases. In doing so, a 69% annotation rate was achieved, bordering the upper limit of that expected for plants without a reference genome^21^. However, a transcript equated with fluorination activity was not detected. Therefore, using the bacterial fluorometabolite biosynthetic genes, we searched the *D. cymosum* transcriptome to find transcripts with homology that were not previously identified. Irrespectively, no significant hit was obtained except for an aldolase and an aldehyde dehydrogenase, and the remaining matches most likely arose from remote sites of homology.

Fluorinases have the exclusive ability to catalyse a C-F bond and thereby facilitate the first step in fluorometabolite biosynthesis^19,27^. At present though, there are only six bacterial fluorinases and none with eukaryotic origin have been found^28–31^. Considering the dissimilarities between bacteria and higher plants, this not only leaves a gap in our knowledge regarding eukaryotic fluorinases, but inherently limits homology searches in higher plants. Zhu et al. also reported that there had been no sequence match between bacterial fluorinases and genomes from the plant kingdom, and suggested the evolution of more than one biological mechanism for fluoride incorporation^32^. While it is possible that the time-points analysed post wounding and the sequencing depth of the *D. cymosum* transcriptome may have been insufficient to detect fluorinase transcripts, the failure to detect any fluorinases may also support the hypothesis of a distinctly different fluorinating enzyme in plants. Consequently, a novel gene for fluorination may be present in this transcriptome, however, remains unidentified.

In contrast to the rarity of fluorinases, aldolases and aldehyde dehydrogenases appear to be more ubiquitous in bacteria, fungi, algae, plants and animals. In *S. cattleya* alone, four putative aldolases were identified. The knockout mutation of one aldolase and in vitro purification of another excluded their involvement in the fluorometabolite pathway^33^. The two remaining aldolases were searched for in the *D. cymosum* transcriptome and one produced a hit with 56% identity. Given that the exact aldolase involved in fluorometabolite biosynthesis remains elusive^33^, it is difficult to decipher if this transcript plays a role in the pathway. The aldehyde dehydrogenase from *S. cattleya*, on the other hand, produced a hit with 41.5% identity in the *D. cymosum* transcriptome. This enzyme was shown to accept substrates other than fluoroacetaldehyde and, moreover, Murphy et al. showed that a yeast aldehyde dehydrogenase was also able to catalyse the conversion of fluoroacetaldehyde to fluoroacetate^34^. Aldolases and aldehyde dehydrogenases are not exclusive to the fluorometabolite pathway^35,36^; therefore it is not surprising that some homology was found with transcripts in the *D. cymosum* transcriptome. Further annotation of these transcripts verified their putative function as an aldolase and aldehyde dehydrogenase. Nonetheless, their particular involvement in the fluorometabolite pathway would need to be confirmed, assuming the pathway follows the same course in plants.

While fluoroacetate is widely believed to constitute a defence mechanism, very little is understood about the underlying wound response in *D. cymosum*. Hence, we aimed to characterise the key responses following mechanical stress. Apart from gene discovery, RNA-seq has emerged as an efficient technology for quantifying relative transcript abundance as its accuracy rivals that of other well established methods, such as microarrays and quantitative polymerase chain reaction (qPCR)^21,37–39^. Nevertheless, to ensure reliable results without the need for further validation, only the most significant changes were reported in this study. Overall, 364 DEGs were found which predominantly corresponded to signal transduction mechanisms, phytohormone regulation, transcription factors and defence-related proteins; demonstrating certain hallmarks of the wound response in *D. cymosum*.

Some of the first molecular events upon mechanical wounding in *D. cymosum* were constituted by Ca^2+^, ROS and phosphorylation-dephosphorylation signalling cascades. These molecules, in conjunction with phytohormones, form part of an interconnected signal transduction mechanism essential in regulating cellular responses upon abiotic stress^40^. In *A. thaliana*, mechanical wounding has been shown to trigger a rapid influx of calcium ions^41^. Calcium ions modulate downstream processes by either stimulating the production of ROS (^1^O_2_, O_2_^−^, OH^−^ and H_2_O_2_)^42^ or through the action of calcium-binding proteins (CaMs, CMLs, CBLs and CDPKs)^40,43^. ROS-scavenging enzymes such as catalase, reticuline oxidase, thioredoxin and glutaredoxin were steadily up-regulated in *D. cymosum*, suggestive of a respiratory burst and subsequent processing of ROS following mechanical stress. ROS has been linked to the activation of hormonal pathways and phosphorylation-dephosphorylation cascades. However, the unabated production of ROS can result in severe oxidative damage to DNA, RNA and proteins. As such, ROS-scavenging enzymes are produced to neutralise elevated levels^40,44^. Likewise, several CaMs and CMLs identified in this study were highly up-regulated as a result of wounding, while a CBL4 protein was down-regulated. CaMs and CMLs perceive stress signals by directly binding Ca^2+^ following the influx^43^. Previous studies have reported that conformational changes in CaMs and CMLs, induced upon Ca^2+^-binding, affects interactions with a complex array of target proteins allowing for appropriate stress responses^45^. The down-regulation of a CBL protein correlates with other studies that have implicated this protein in osmotic stress rather than mechanical stress^46,47^. CaMs and CMLs are also independently capable of activating phosphorylation-dephosphorylation cascades to elicit a defence response. Numerous kinases and phosphatases which modulate responses through phosphorylation patterns were found in the present study. However, among the most notable were MAP kinases. Both MAPKKK1 and MAPK3, associated with stress responses in *A. thaliana*^48,49^, were persistently up-regulated in *D. cymosum*. Similar signalling responses have been observed in *A. thaliana*^50^. Moreover, Walley et al. went on to show that these responses (Ca^2+^, MAPK and WRKY) are highly up-regulated in *A. thaliana* just five minutes after mechanical wounding^50^. There is also evidence for extensive cross-talk between these signalling cascades and phytohormone responses^40^.

Phytohormones regulate a variety of plant processes such as growth, development and defence or tolerance to abiotic and biotic stress. The influence of these molecules on various stresses has been widely studied^51^. The findings in this study demonstrated that the JA, SA and ET pathways in *D. cymosum* were up-regulated in response to wounding while the IAA, GA and ABA pathways were down-regulated. The IAA and GA pathways are typically involved in plant development^52^. But upon wounding, there is a molecular reconfiguration to alter from a growth state to a defence state, therefore, it is not unusual to see a reduction in growth related hormones. The ABA pathway is associated with seed dormancy and stomatal closure, implicating this phytohormone in drought and osmotic stress responses^53^. In contrast, although also involved in developmental processes; the JA, SA and ET pathways have more frequently been affiliated with defence regulation in plants^52^. The JA pathway, which plays the most conspicuous role, is linked to tissue damage caused by mechanical wounding, insects, herbivores and necrotrophic pathogens^51^. The detection of both early (13-LOX) and late (OPR3) enzymes involved in the biosynthesis of JA in *D. cymosum* suggested the rapid response of JA to mechanical wounding. In addition, several JAZ proteins were also found to be up-regulated, most predominantly at T:60. Under normal conditions, JAZ proteins regulate JA-responses by acting as repressors of MYC transcription factors. However, following wounding, there is rapid synthesis of JA. Jasmonates have been shown to bind the receptor, CORONATINE INSENSITIVE 1 (COI1), complexed with JAZ. This targets JAZ for degradation and releases MYC allowing for transcription of wound-responsive genes. The interaction between JA and JAZ provides tight regulation of responses, however, the excessive amplification of JAZ proteins also suggested a decline in the JA-pathway at T:60^51^. Interestingly, this is when SA started to show some activity in *D. cymosum*, evident by the down-regulation of EDR2. The antagonistic relationship between JA and SA has been reported and the findings here are coherent with other studies^54,55^. Similar to *D. cymosum*, Lee et al. showed a rapid increase in JA levels 30 min after mechanical wounding in *Oryza sativa* leaves. This JA-burst also coincided with a decline in SA levels^55^. SA is typically activated in response to biotrophic pathogens and mediates pathogen resistance^53^. Given that mechanical wounding not only damages plant tissue, but also provides a potential entry point for pathogens, it is not surprising to see SA up-regulated at a later time point in *D. cymosum*. Again, detection of both early (SAM2) and late enzymes (ACO) involved in the biosynthesis of ET^56^ in *D. cymosum* suggested activity of the pathway. However, in contrast to SA, ET is known to operate synergistically with JA to activate expression of defence genes^52,53^. Cross-talk between phytohormone signalling pathways (JA, SA and ET) is a mechanism that effectively allows for the regulation of stress response. This regulation primarily occurs at the level of transcription^41^.

Transcription factors constituted one of the largest groups of genes differentially expressed in *D. cymosum* following mechanical stress. Transcription factors act directly on stress-responsive genes, such as defence related genes or secondary metabolite biosynthetic genes, and are thus one of the most crucial aspects in mounting a tailored response^57^. In the present study, WRKY transcription factors were most prominently up-regulated. WRKY transcription factors are commonly associated with a variety of stress responses^58^. WRKY15, WRKY18, WRKY33 and WRKY46 particularly, have been associated with pathogen infection, drought stress, osmotic stress and mechanical wounding^59–65^. In addition, ERFs are the major target of ET signalling and act on a variety of defence related genes. Secondary metabolites from the phenylpropanoid and terpenoid pathways have been shown to play roles in stress response^66–69^. Here, only the terpenoid pathway was found amongst the most differentially regulated suggesting an important role for terpenoid metabolism in the wound response in *D. cymosum*.

## Conclusions

*D. cymosum* is a significant part of history representing the source of the first fluorinated metabolite. In the present study, a comprehensive transcriptome of *D. cymosum* was produced. To the best of our knowledge, this is the first transcriptome from *Dichapetalaceae*, a family comprising of several FA-producing plants. The gene expression profiles of *D. cymosum* were evaluated in response to wounding. This revealed the fine-tuning of signalling cascades, phytohormone regulation, transcription factors and secondary metabolites most important in the wound response. However, the role of FA in wound responses remains unclear. The transcriptomic data produced in this study has been made available and will have a significant impact on future research into the origin of FA in plants.

## Methods

### Collection of plant material and mechanical wounding

*Dichapetalum cymosum* (gifblaar) samples were collected at the Pretoria National Botanical Gardens, South Africa, in September 2015 during the spring season. In order to assess the genes associated with a wound response, mechanical wounding studies were performed according to Reymond et al.^22^. An initial sample of the unwounded leaf tissue was taken at time zero (T:0) representing the control. Leaves, still attached to the plant, were then wounded. Approximately 40% of the leaf surface was punctured using sterile forceps. Leaves were severed from the plant at 30 min (T:30) and 60 min (T:60) post wounding. Technical replicates of samples at each time point were immediately frozen in liquid nitrogen and later transferred to a − 80 °C freezer until further processing.

### RNA isolation, construction of cDNA libraries and Illumina sequencing

Total RNA was isolated based on the method outlined by Xu et al.^70^. Total RNA was quantified using the Qubit Fluorometer 2.0 (Life Technologies) and RNA integrity was assessed using 1% agarose gel electrophoresis. cDNA library construction and Illumina sequencing were performed at the Agricultural Research Council (Pretoria, South Africa) as a commercial service. Ribosomal RNA was depleted using the RiboZero Plant rRNA Removal Kit (Epicentre). Thereafter, cDNA libraries for each time point (in triplicate) were created individually using the TruSeq Stranded mRNA Library Preparation Kit (Illumina). Paired-end sequencing was performed for each of the time-point samples and their respective replicates on the HiSeq 2500 (Illumina) platform using the HiSeq Reagent Kit v4 (Illumina).

### Quality control and de novo assembly using Trinity

The web links for all tools and databases used in this study are provided in Supplementary Table S4. The quality of each library, before and after trimming, was analysed using FastQC version 0.11.2^71^. All adaptor sequences and short sequences (< 40 bp) were trimmed using Trimmomatic (version 0.30)^72^. Only the paired reads whereby both the forward and reverse read survived the processing were used for downstream analysis. Prior to the transcriptome assembly, all forward reads across all time points (T:0, T:30 and T:60) and replicates were combined into one file. The same was done for the reverse reads into a separate file to create a single RNA-seq data set. Using the combined forward and reverse reads, a comprehensive reference transcriptome (T:0 + T:30 + T:60) was assembled de novo using Trinity (version 2.0.2) under default settings^73^. The completeness of the assembled transcriptome was assessed using BUSCO (version 3.1.0) based on the eudicotyledons lineage dataset containing 2121 benchmarked orthologues (n: 2121).

### Annotation of the *D. cymosum* transcriptome

In order to assign functional descriptions, Trinity transcripts were queried against the UniProt (SwissProt and TrEMBL), Gene Ontology (GO) and the evolutionary genealogy of genes: Non-supervised Orthologous Groups (eggNOG) databases using a blastx search with an e-value cut-off of 1E-05^74–76^. For GO, a set of plant-specific GO Slims were used to classify the annotations into 97 terms under the molecular function, cellular component and biological process categories. Annotations from eggNOG were allocated single-letter codes based on Clusters of Orthologous Groups (COG) to categorise descriptions into 25 functional groups. The Kyoto Encyclopedia of Genes and Genomes (KEGG) database was used to map pathways represented in the *D. cymosum* transcriptome^77^. This was performed using the web server, KOBAS 3.0^78^.

Simple Sequence Repeats (SSR’s) were identified in assembled transcripts using the Microsatellite (MISA) identification tool^79^. Search parameters included di-, tri-, tetra-, penta-, and hexa-nucleotide sequences that were repeated a minimum of ten, seven, five, five and five times respectively. The maximum number of bases allowed interrupting 2 SSR’s was set at 100 bp.

TransDecoder (version 2.0.1) was then used to identify open reading frames (ORFs) with a minimum length of 100 consecutive amino acids from the assembled transcripts. HMMER (version 3.1b1) was used to scan the protein predictions for the presence of conserved domains against the Pfam database^80^. Additionally, protein sequences were queried against the ExPASy-Enzyme database to assign EC numbers^81^, and against the Plant Transcription Factor Database (PlantTFDB) to identify transcription factors^82^. The e-value cut-off for blastp searches was 1E-05.

### Investigating the *D. cymosum* transcriptome for putative fluorometabolite biosynthetic genes

The translated *D. cymosum* transcriptome, in all six frames, was searched for putative enzymes involved in fluorometabolite biosynthesis using blastp. Enzymes involved in fluorometabolite production in *S. cattleya* were obtained from GenBank and used as the query sequences. This included the fluorinase (WP_014144878.1), a purine nucleoside phosphorylase (PNP) (WP_014144879.1), an isomerase (WP_014142773.1), two aldolases (WP_014141855.1, WP_014150905.1), an aldehyde dehydrogenase (WP_014141713.1) and a 4-fluorothreonine transaldolase (WP_014151017.1). The best hit in the *D. cymosum* transcriptome for each of the queried enzymes was extracted and searched against the non-redundant (nr) database using blastp to obtain putative functions.

### Abundance estimation and differential expression analysis

Abundance estimation and differential expression analysis were performed according to Haas et al. using software included in the Trinity (version 2.0.2) package^73^. In order to assess differentially expressed genes (DEGs) in response to wounding, the high-quality clean reads from each library within time points T:0, T:30 and T:60 were individually aligned to the assembled reference transcriptome (described in ‘Quality control and de novo assembly using Trinity’) using Bowtie. The relative abundance of each transcript was estimated using RNA-Seq by Expectation Maximization (RSEM) and differential expression analysis was conducted using edgeR. Expression values were normalised using the trimmed mean of *M*-values (TMM) method to account for inherent biases introduced through variations in sample composition and sequencing depth. DEGs with normalised expression values that adhered to a False Discovery Rate (FDR) of ≤ 0.001 and showed a ≥ fourfold change between at least two time points were selected. This allowed for reliable estimations of relative levels which can be used to directly compare differences in gene expression between samples.

### Annotation of DEGs

DEGs were searched against the SwissProt database and assigned GO terms using Function Annotator^83^. REVIGO was used to enrich GO processes associated with DEGs^84^. KEGG pathways were assigned using KOBAS version 3.0 and the PlantTFDB was used to identify transcription factors associated with the regulation of a wound response.

## Supplementary information

Supplementary Information.

## Data Availability

The raw sequencing reads generated in this study have been deposited onto the NCBI Sequence Read Archive (SRA) under the umbrella BioProject Accession No. PRJNA634987.
