# Supplementary material for: Functional characterisation of the transcriptome from leaf tissue of the fluoroacetate-producing plant, Dichapetalum cymosum, in response to mechanical wounding
Source: Sci Rep. 2020 Nov 25;10:20539. doi: 10.1038/s41598-020-77598-7 (PMC7688953; doi:10.1038/s41598-020-77598-7)
Supplement: Supplementary file 1 — Supplementary Information. [file 41598_2020_77598_MOESM1_ESM.pdf]

Functional characterisation of the transcriptome from leaf tissue of the fluoroacetate-producing plant, *Dichapetalum cymosum*, in response to mechanical wounding

Selisha A. Sooklal[1,+] , Phelelani T. Mpangase[2,+] , Mihai-Silviu Tomescu[1], Shaun Aron[2] , Scott Hazelhurst[2] , Robert H. Archer[3] & Karl Rumbold[1,*]

[1]Department of Microbiology, School of Molecular and Cell Biology, University of the Witwatersrand, Johannesburg, 2000, South Africa

[2]Sydney Brenner Institute for Molecular Biosciences, University of the Witwatersrand, Johannesburg, 2000, South Africa

[3]National Herbarium, South African National Biodiversity Institute, Pretoria, 0186, South Africa

[*]Corresponding author e-mail: karl.rumbold@wits.ac.za

[+]These authors contributed equally to this work

Supplementary Information

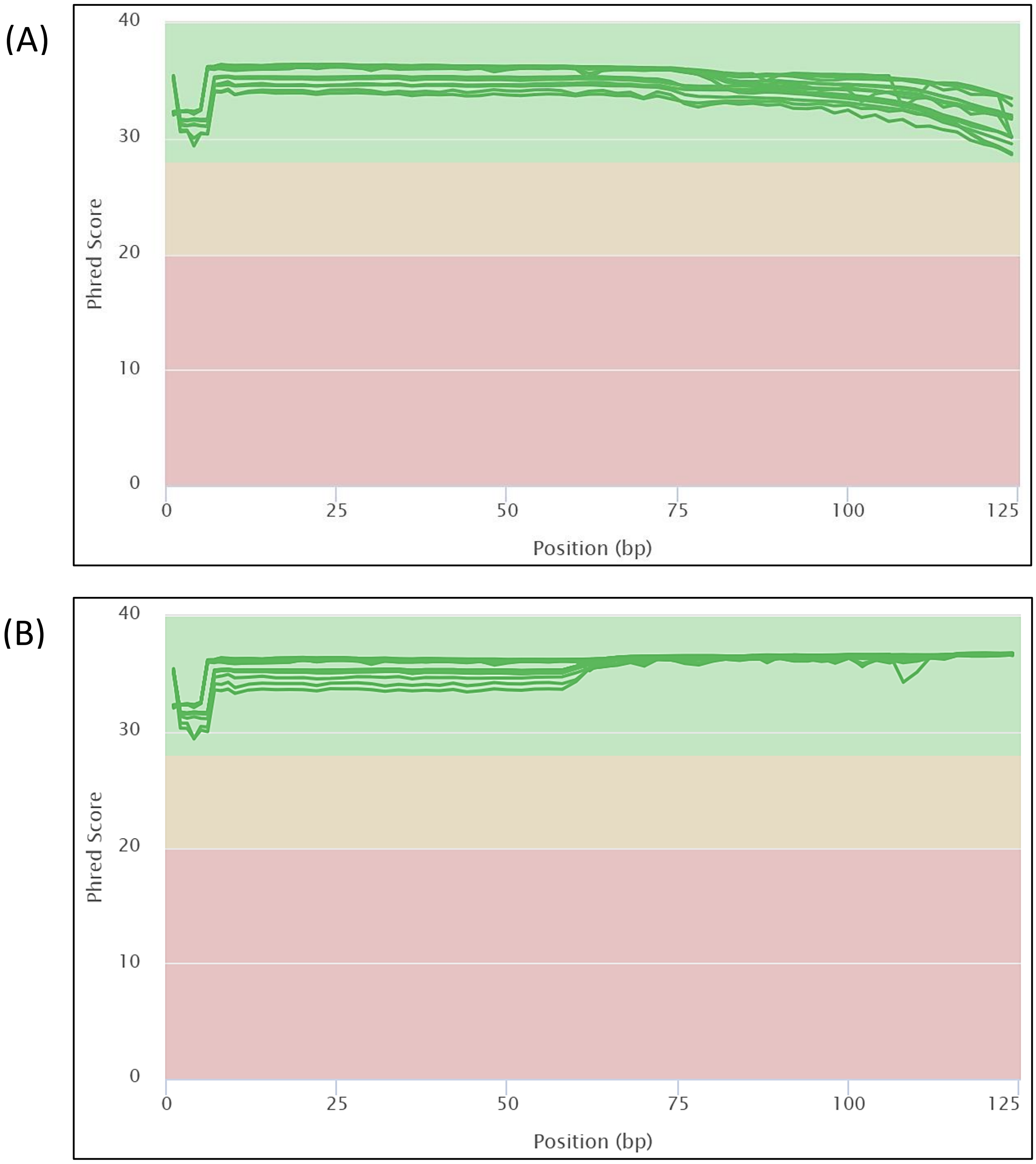

Figure S1. MultiQC report showing the Phred score for each sequenced library of the unwounded and wounded *D. cymosum* leaves **(A)** before trimming and **(B)** after trimming using Trimmomatic.

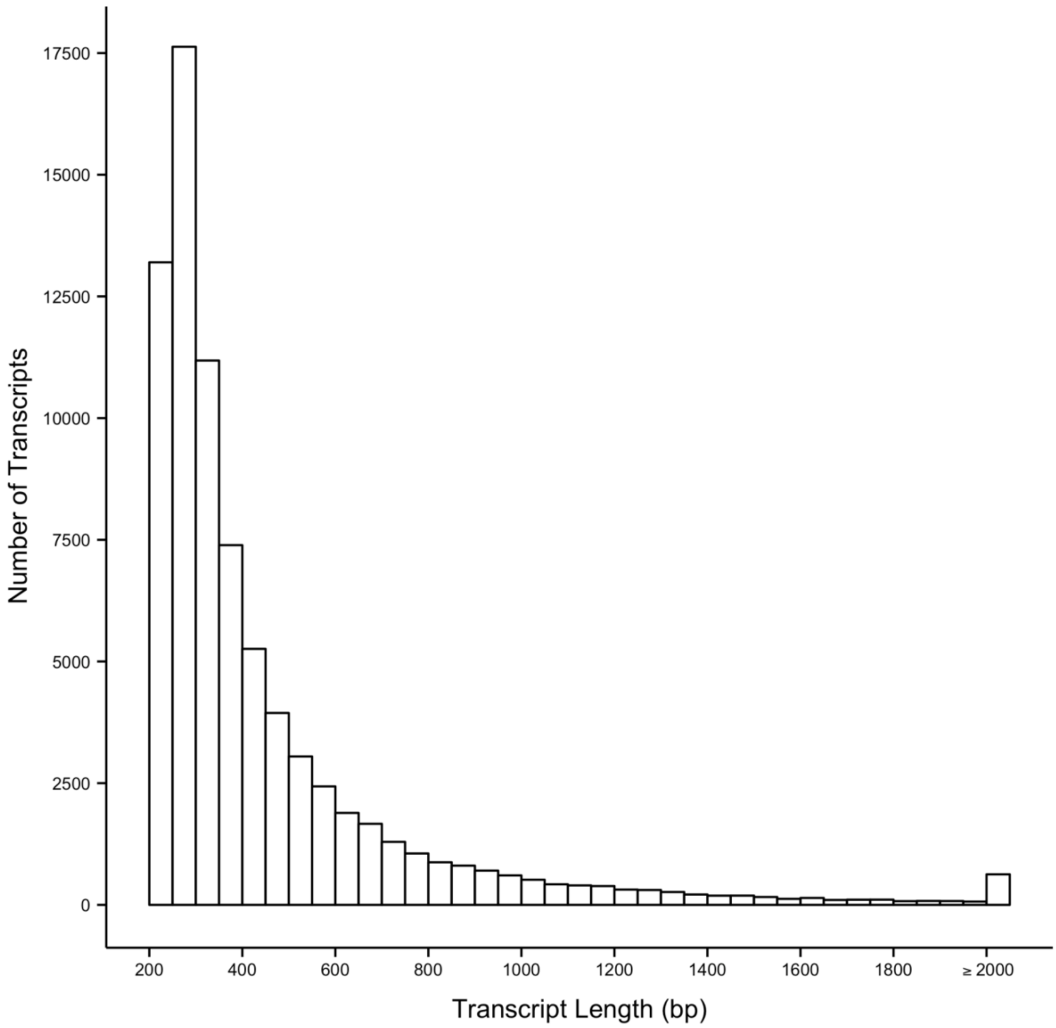

Figure S2. Length distribution of the assembled transcripts. The 77,845 transcripts assembled with Trinity had an average length of 454 bp. Majority of transcripts fell between 200-500 bp while the shortest transcript had a length of 224 bp and the longest transcript had a length of 10,795 bp.

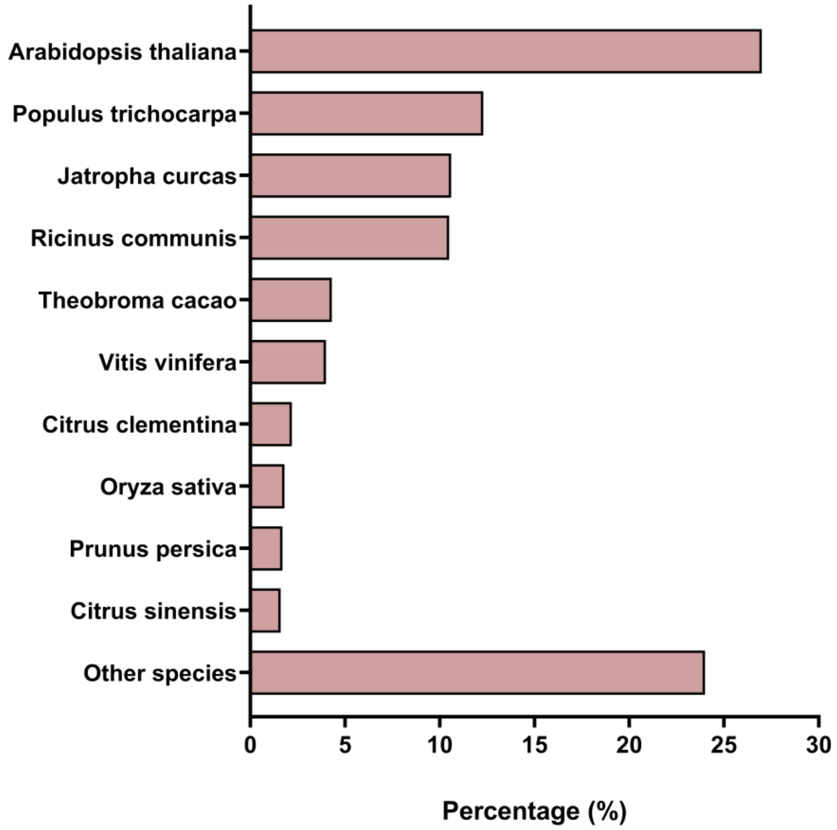

Figure S3. Species distribution of annotations obtained from the *D. cymosum* transcriptome. Majority of the *D. cymosum* transcripts annotated by the UniProt databases exhibited homology to sequences from *Arabidopsis thaliana*.

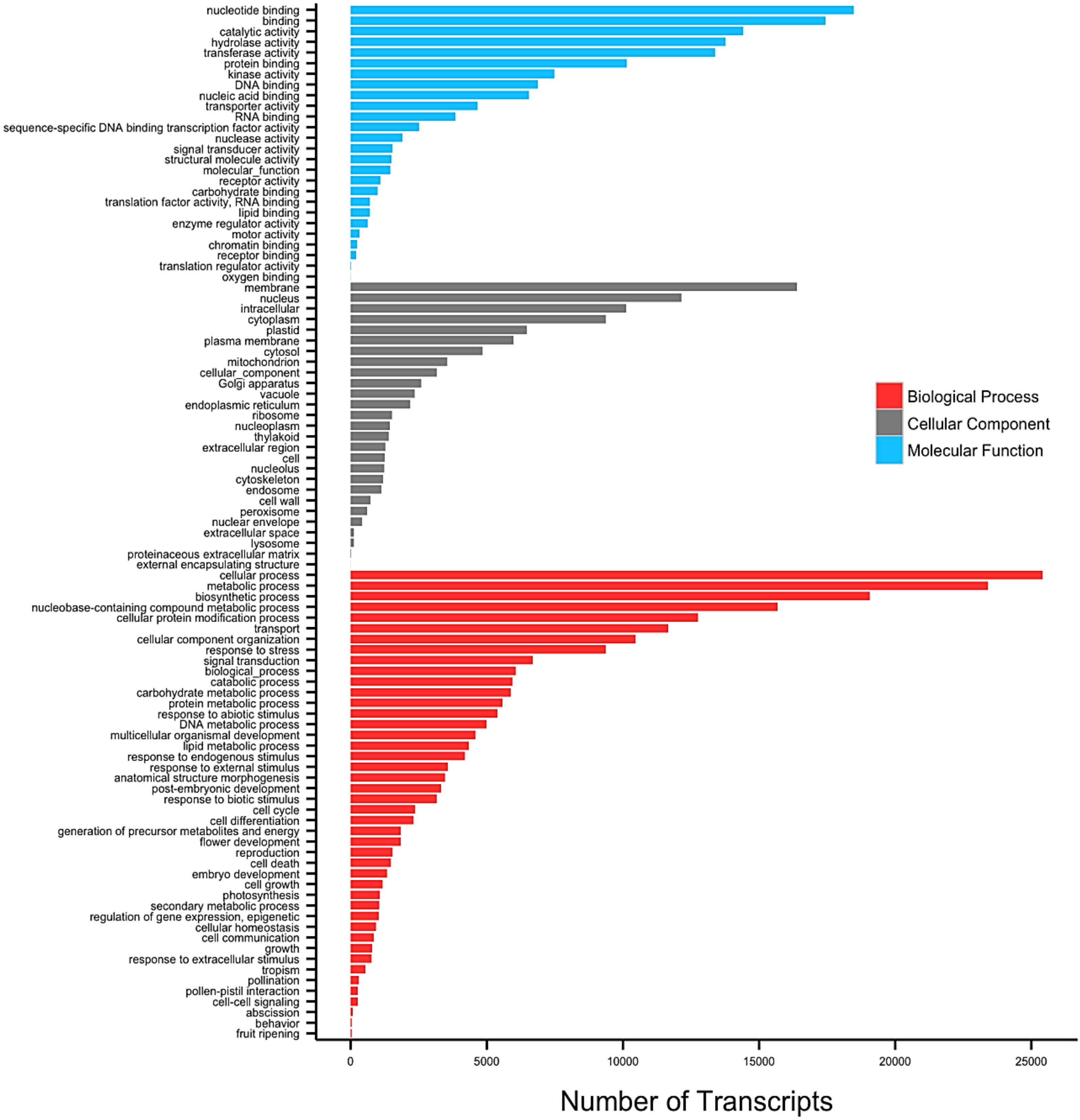

Figure S4. GO classification of the *D. cymosum* transcriptome. GO classified transcripts into three broad domains: biological process (red), cellular component (grey) and molecular function (blue). Annotations were further sub-divided using 97 plant specific GO Slims (listed on the y-axis).

Table S1. Detection of SSR's in the *D. cymosum* transcriptome. SSR's were found using the MISA tool.

	Number of SSR's	Percentage of total SSR's	Most frequent repeat
**di-**	393	25.6	AG/CT
**tri-**	887	57.8	CTT/AAG
**tetra-**	179	11.7	AAAT/ATTT
**penta-**	50	3.3	AGTTT/AAACT
**hexa-**	25	1.6	TGTGGC/GCCACA

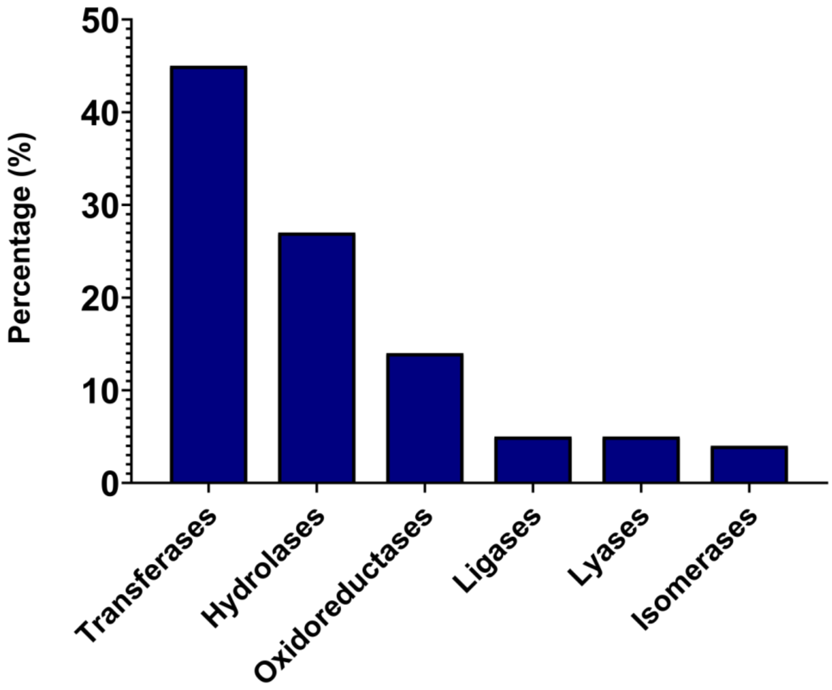

Figure S5. EC classification of 7,311 *D. cymosum* transcripts into the oxidoreductase (14%), transferase (45%), hydrolase (27%), lyase (5%), isomerase (4%) and ligase (5%) categories.

Table S2. Blastp results following the query of the *S. cattleya* fluorometabolite biosynthetic genes against the *D. cymosum* transcriptome.

Query ID	Hit in transcriptome (Subject ID)	% of identical matches	Alignment length	No. of mis-matches	No. of gap openings	Start-end of alignment in query	Start-end of alignment in subject	e-value	Bit score	Putative function of *D. cymosum* transcript % identity, query coverage, e-value
WP_014144878.1 **Fluorinase**	TR6001_c0_g2_i4_(-2)	36.8	38	23	1	155-192	36-72	5.6	29.3	**Ceramide inositol-phosphotransferase** 93.0%, 73%, 7e-40
WP_014144879.1 **PNP**	TR35484_c0_g1_i1_(-2)	29.6	54	35	1	167-220	1-51	1.4	31.2	**LRR receptor-like serine/threonine-protein kinase** 76.6%, 100%, 1e-50
WP_014142773.1 **Isomerase**	TR31930_c0_g1_i1_(-2)	26.5	253	174	6	84-333	172-415	3.91E-12	69.3	**IF-2B domain-containing protein** 90.9%, 77%, 0.0
WP_014141855.1 **Aldolase**	TR16309_c0_g2_i1_(-1)	55.9	256	110	2	112-367	2-254	3.17E-81	252	**Uncharacterised protein: transaldolase family** 92.6%, 85%, 9e-169
WP_014150905.1 **Aldolase**	TR25733_c0_g1_i1_(-2)	38.4	99	59	1	45-143	48-144	5.22E-15	73.6	**Uncharacterised protein: putative dehydrogenase** 79.3%, 72%, 2e-62
WP_014141713.1 **Aldehyde dehydrogenase**	TR21022_c0_g1_i3_(-1)	41.5	482	259	10	24-496	114-581	1.64E-112	348	**Aldehyde dehydrogenase family 2 member** 85.2%, 86%, 0.0
WP_014151017.1 **4-FT transaldolase**	TR44173_c0_g2_i1_(-1)	21.7	423	270	11	17-395	41-446	1.93E-13	75.1	**Serine hydroxymethyltransferase** 87.3%, 100%, 0.0

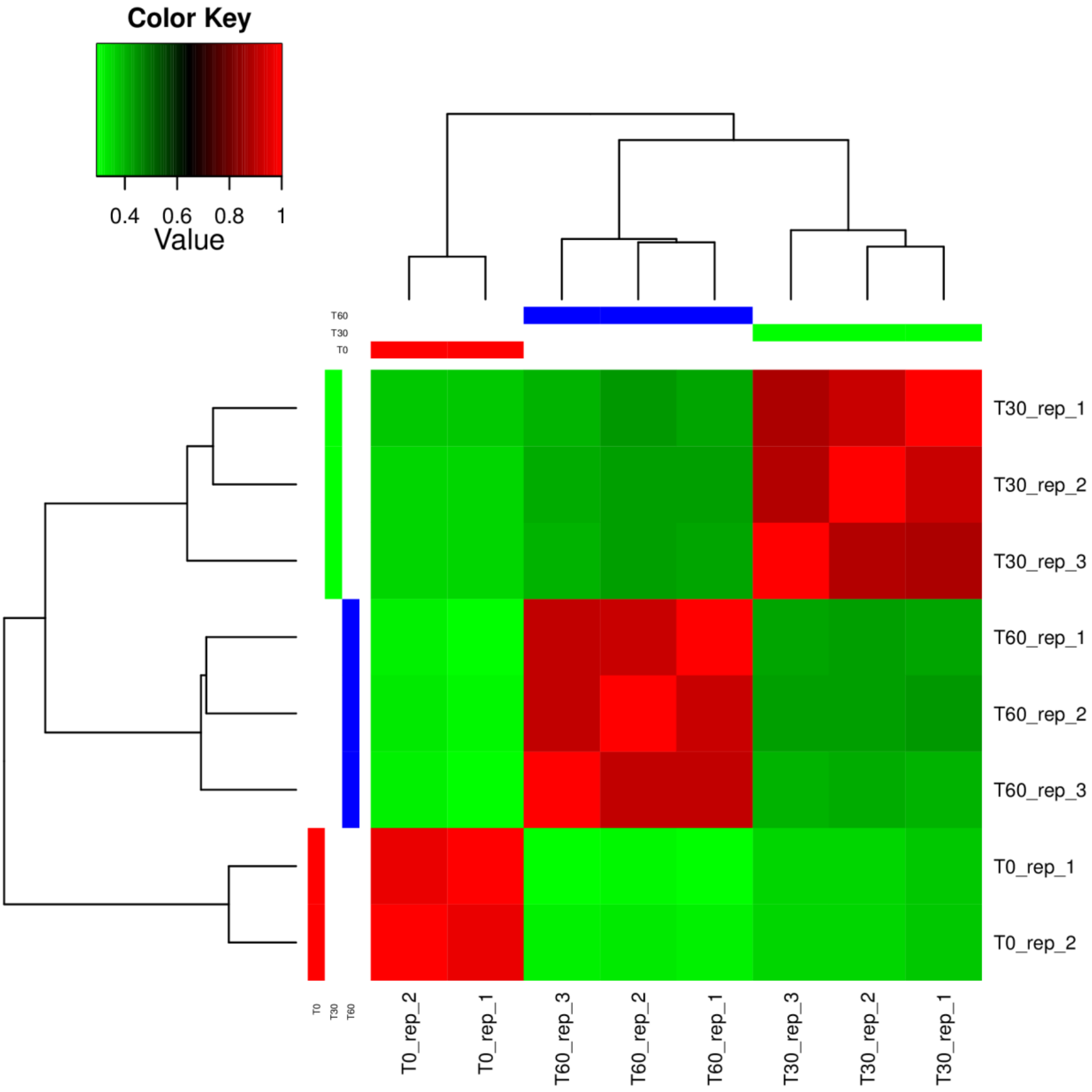

Figure S6. Correlation matrix between replicates of each time point. A perfect correlation (1) is indicated in red while lower correlation (0.4) is indicated in green. Replicates within each time point shows good correlation indicating reproducibility of experimental data.

Table S3. The regulation of transcripts in response to mechanical wounding in *D. cymosum*

Category	Putative fuction	Regulation			Transcript ID
		T:0	T:30	T:60	
**Transcription factors**					
**B3**	Auxin response factor 11	↑	↓	↑	TR9948_c0_g1_i2
**bHLH**	Transcription factor bHLH13	↓	↑	↑	TR43985_c0_g2_i1
	Transcription factor bHLH148	↑	•	↓	TR44545_c0_g1_i1
**C2H2**	Zinc finger protein ZAT10	↓	↑	•	TR31532_c0_g1_i1
**C3H**	Zinc finger CCCH domain−containing protein 29	↓	↑	↑	TR1170_c0_g1_i1<br>TR1170_c0_g1_i2<br>TR6469_c1_g1_i1
	Zinc finger CCCH domain−containing protein 47	↓	↑	↑	TR9322_c0_g1_i1
	Zinc finger CCCH domain−containing protein 33	↓	↑	↑	TR861_c0_g2_i3
**CAMTA**	Calmodulin−binding transcription activator 2	↓	•	↑	TR2498_c0_g2_i4
**Co-like**	Zinc finger protein CONSTANS−LIKE 16	↑	↑	↓	TR25717_c0_g1_i1<br>TR25717_c0_g2_i1
**Dof**	Cyclic dof factor 3	↑	↑	↓	TR25324_c0_g1_i1
**ERF**	Ethylene−responsive transcription factor RAP2−4	↓	↑	↑	TR10154_c3_g2_i1
	Ethylene−responsive transcription factor ERF109	↓	↑	↑	TR10345_c0_g1_i1
	Ethylene−responsive transcription factor ERF105	↓	↑	↑	TR28723_c1_g1_i1
**GRAS**	Chitin−inducible gibberellin−responsive protein 1	↓	↑	•	TR31813_c1_g1_i1
	Scarecrow−like protein 14	↓	↑	↓	TR42270_c0_g1_i1<br>TR680_c0_g1_i1
**MIKC_MADS**	MADS−box protein SVP	↑	↑	↓	TR14910_c0_g1_i3
**M-type_MADS**	MADS−box protein JOINTLESS	↑	↑	↓	TR8542_c0_g4_i7
**MYB**	Myb−related protein 306	↑	↑	↓	TR34592_c0_g1_i1
**MYB_related**	Protein REVEILLE 1	↑	•	↓	TR39440_c1_g1_i2
**NAC**	NAC transcription factor ONAC010	↓	↑	•	TR11949_c0_g1_i2
	NAC domain−containing protein 78	↓	↑	↑	TR24150_c0_g1_i3
	NAC domain−containing protein 86	↓	↑	•	TR6271_c0_g2_i4
**NF-YC**	Nuclear transcription factor Y subunit C−2	↓	↓	↑	TR30184_c0_g1_i1
**Trihelix**	Trihelix transcription factor ASIL2	↑	•	↓	TR45736_c0_g1_i1
**WRKY**	Probable WRKY transcription factor 53	↓	↑	•	TR1133_c1_g1_i1<br>TR1133_c1_g1_i2<br>TR1133_c1_g1_i3
	WRKY transcription factor 6	↓	↑	↑	TR21652_c0_g1_i3
	Probable WRKY transcription factor 33	↓	↑	•	TR27647_c4_g1_i3
	Probable WRKY transcription factor 46	↓	↑	↑	TR31296_c0_g1_i4<br>TR31296_c0_g1_i5<br>TR34179_c0_g1_i3
	Probable WRKY transcription factor 15	↓	↑	↑	TR41435_c3_g1_i2
	WRKY transcription factor 18	↓	↑	↑	TR43264_c1_g1_i1
**Phytohormone responses**					
**Jasmonates**	Linoleate 13S−lipoxygenase	↓	↑	↑	TR33072_c0_g2_i1
	12−oxophytodienoate reductase 3	↓	↑	↑	TR37738_c0_g1_i5<br>TR37738_c0_g1_i2
	Protein TIFY 6B (JAZ3)	↓	↓	↑	TR38457_c1_g1_i1
	Protein TIFY 10A (JAZ1)	↓	↑	↑	TR31372_c1_g2_i2<br>TR24873_c0_g1_i1<br>TR24873_c0_g1_i3
	Protein TIFY 11B (JAZ6)	↓	↑	↑	TR30411_c0_g2_i1
**Ethylene**	*S*-adenosylmethionine synthase 2	↓	↑	↑	TR6199_c0_g1_i1<br>TR6199_c0_g1_i2
	1−aminocyclopropane−1−carboxylate oxidase	↓	↑	↑	TR22364_c0_g1_i2

**Gibberellins**	Gibberellin 2–beta–dioxygenase 2	↓	↑	↓	TR3986_c1_g1_i1
	Gibberellin receptor GID1	↑	↑	↓	TR5384_c0_g1_i1
	DELLA protein GAI	↑	↓	•	TR37799_c0_g1_i1
**Auxins**	IAA–amino acid hydrolase ILR1–like 4	↓	↑	↑	TR44491_c0_g1_i1
	Auxin–repressed 12.5 kDa protein	↑	↑	↓	TR29771_c0_g3_i1
	Auxin-responsive protein IAA16	↑	•	↓	TR17243_c0_g1_i1
	Tryptophan aminotransferase-related protein 1	↑	↓	↓	TR12889_c0_g1_i1<br>TR12889_c0_g1_i2
	Tryptophan aminotransferase-related protein 1	↑	↑	↓	TR38367_c11_g13_i2<br>TR38367_c11_g20_i1<br>TR8487_c0_g2_i4<br>TR23600_c0_g2_i1
**Salicylic acid**	Protein ENHANCED DISEASE RESISTANCE 2	↑	↑	↓	TR43024_c0_g1_i10
**Abscisic acid**	Abscisic stress–ripening protein 3	↑	•	↓	TR21333_c0_g1_i1
**Heat shock proteins**					
	Heat shock 70 kDa protein 2	↓	•	↑	TR39531_c1_g1_i1
	Heat shock 70 kDa protein 4	↓	•	↑	TR24193_c0_g1_i1
**MAP kinases**					
	Mitogen–activated protein kinase kinase kinase MPK1	↓	↑	↑	TR32256_c0_g1_i1
	Mitogen-activated protein kinase 3	↓	↑	•	TR41832_c1_g1_i1<br>TR41832_c1_g1_i2<br>TR41832_c1_g1_i3
**Secondary metabolite biosynthesis**					
**Terpenoid biosynthesis**	1–deoxyxylulose–5–phosphate synthase	↓	↓	↑	TR36209_c0_g2_i1
	Isoprene synthase	↓	↓	↑	TR25944_c2_g1_i2<br>TR25944_c2_g1_i8
**Ca^{2+} signalling**					
	Calcium–transporting ATPase 1	↓	↑	↑	TR12376_c2_g6_i2
	Calcium–transporting ATPase 2	↓	↑	↑	TR12376_c1_g1_i1<br>TR12376_c1_g1_i2
	Calcium–transporting ATPase 12	↓	↑	↑	TR5940_c0_g1_i1
	Calmodulin-like protein CML45	↓	↑	•	TR37317_c0_g1_i1
	Calmodulin-like protein CML27	↓	↑	↑	TR24640_c0_g1_i2
	Calmodulin (CaM)	↓	↑	↑	TR4728_c0_g1_i2
	Calmodulin–1 (CaM–1)	↓	↑	•	TR36505_c0_g1_i2
	Calmodulin–binding transcription activator 4	↓	•	↑	TR25454_c0_g2_i1
	Calcineurin B–like protein 4	↑	•	↓	TR18111_c0_g2_i1
	Multiple C2 and transmembrane domain–containing protein 1	↓	↑	↑	TR13280_c0_g1_i1
**Transporters**					
	Boron transporter 4	↓	↓	↑	TR5388_c1_g1_i8
	Copper transport protein ATX1	↑	↓	↓	TR36133_c0_g1_i2<br>TR36133_c0_g1_i1
	Probable aquaporin PIP1–2	↓	↑	↑	TR27512_c4_g1_i1
	Probable inorganic phosphate transporter 1–5	↓	↑	↑	TR41082_c0_g1_i2
	Vacuolar amino acid transporter 1	↓	↑	↑	TR13739_c0_g1_i3<br>TR13739_c0_g1_i4
	Protein TRANSPARENT TESTA 12	↓	↑	↑	TR22329_c0_g1_i2
**Proteolysis**					
	E3 ubiquitin–protein ligase PUB23	↑	↓	↓	TR16704_c0_g1_i1
	E3 ubiquitin–protein ligase RING1–like	↓	↑	↓	TR42553_c0_g1_i1
	Probable E3 ubiquitin–protein ligase HERC1	↑	↑	↓	TR16704_c0_g1_i1
	Polyubiquitin 11	↓	•	↑	TR30506_c1_g1_i5

					TR30506_c1_g1_i6<br>TR7579_c0_g3_i2
	Ubiquitin carboxyl−terminal hydrolase 12	↓	↑	↓	TR12222_c1_g1_i2
	Putative U−box domain−containing protein 42	↓	↑	↑	TR33493_c0_g1_i1
	Aspartic proteinase A1	↑	•	↓	TR39950_c0_g2_i1
	Aspartic proteinase−like protein 1	↓	↑	↑	TR21898_c0_g1_i3
	ATP−dependent zinc metalloprotease	↓	↑	↓	TR7959_c0_g2_i1
	Basic secretory protease	↓	↑	↑	TR7316_c1_g1_i1
	Thiol protease aleurain	↑	↑	↓	TR39522_c0_g3_i1
	Rhomboid protease GluP	↑	↓	↓	TR35072_c0_g1_i1
**Mitochondrial respiratory chain**					
	NADH dehydrogenase subunit 1	↓	↑	↓	TR23950_c0_g1_i1
	NADH dehydrogenase subunit 2	↓	↑	↓	TR25849_c0_g1_i1<br>TR6136_c0_g1_i1<br>TR12975_c2_g5_i5<br>TR12975_c2_g9_i1
	NADH dehydrogenase subunit 3	↓	↑	↓	TR4733_c0_g1_i1
	NADH dehydrogenase subunit 4	↓	↑	↓	TR13996_c0_g1_i1
	NADH dehydrogenase subunit 5	↓	↑	↓	TR39326_c0_g1_i1
	NADH dehydrogenase subunit 6	↓	↑	↓	TR10815_c0_g1_i1
	NADH dehydrogenase subunit 7	↑	↓	•	TR742_c2_g1_i1<br>TR13536_c2_g2_i4
	Cytochrome c oxidase subunit 1	↓	↑	↓	TR13956_c0_g1_i1
	Cytochrome c oxidase subunit 2	↓	↑	↓	TR6576_c0_g1_i1
	Cytochrome c oxidase subunit 3	↓	↑	↓	TR44521_c0_g1_i1
	Cytochrome b	↓	↑	↓	TR45100_c0_g1_i1
**Photorespiration**					
	Cytochrome b6	↓	↑	↑	TR33038_c0_g1_i1<br>TR10321_c1_g1_i1
	Cytochrome b6-f complex subunit 8	↓	↑	↑	TR30276_c0_g1_i1
	Ferredoxin-2	↓	↑	↑	TR38279_c0_g1_i1
	Photosystem II protein D1	↓	↑	↑	TR45648_c2_g1_i1<br>TR45648_c0_g2_i1
	Photosystem II D2	↓	↓	↑	TR33459_c0_g3_i2
	Photosystem II CP47 reaction centre protein	↓	↑	↓	TR2524_c1_g1_i1
	Photosystem II reaction centre protein H	↓	↑	↑	TR10321_c0_g1_i1
	ATP synthase	↓	↑	↑	TR38824_c0_g1_i2<br>TR26918_c2_g5_i1
	Photosystem I assembly protein Ycf3	↓	↑	↑	TR9274_c0_g2_i2
	Tetrapyrrole−binding protein	↑	↓	↓	TR10917_c0_g1_i1
**Oxidative stress**					
	Catalase isozyme 2	↓	↑	↑	TR19830_c0_g1_i1
	Superoxide dismutase	↑	↓	↓	TR39709_c0_g1_i1
	Glutaredoxin−C9	↓	↑	•	TR5452_c0_g1_i1
	Reticuline oxidase−like protein	↓	↑	↑	TR37436_c0_g1_i1
	Thioredoxin F−type	↓	↓	↑	TR43948_c1_g1_i4
	NAD(P)H-quinone oxidoreductase	↓	↑	↑	TR37887_c0_g1_i1
**Phosphatases**					
	Purple acid phosphatase 3	↓	↑	↑	TR40803_c0_g2_i6
	Probable protein phosphatase 2C 24	↑	•	↓	TR44379_c0_g1_i2
	Probable protein phosphatase 2C 25	↓	↑	•	TR31269_c0_g1_i1
	Protein phosphatase 2C 37	↑	↑	↓	TR26183_c0_g1_i1
	Probable protein phosphatase 2C 44	↓	↑	↓	TR5165_c0_g2_i2
	Probable protein phosphatase 2C 63	↓	↑	↑	TR33687_c0_g1_i9

**Pathogenesis-related**					
	Protein argonaute 5	↓	•	↑	TR19756_c0_g1_i1
	Probable disease resistance protein	↓	↑	•	TR28205_c1_g1_i1<br>TR28205_c1_g1_i2
	Indole−3−acetic acid−induced protein ARG2	↓	↑	•	TR20755_c0_g1_i1
	MLP−like protein 31	↓	↑	↑	TR39328_c0_g1_i1
	Pleiotropic drug resistance protein 2	↓	↑	↓	TR42006_c0_g2_i4<br>TR34682_c0_g1_i3
	Pleiotropic drug resistance protein 2	↓	↑	↑	TR42006_c0_g2_i2<br>TR34682_c0_g1_i2
	Putative late blight resistance protein homolog R1B−19	↓	↑	↓	TR22540_c0_g1_i2
**Cell wall – signalling and modification**					
	Remorin (pp34)	↑	↑	↓	TR10463_c1_g1_i2
	Protein EXORDIUM	↓	↑	•	TR29005_c0_g1_i1
**Other kinases**					
	Probable receptor−like protein kinase	↓	↑	↓	TR7041_c0_g1_i1<br>TR7041_c0_g1_i2
	Serine/threonine−protein kinase prp4	↑	•	↓	TR7054_c0_g1_i1
	Leucine−rich repeat receptor−like serine/threonine/tyrosine−protein kinase	↓	↑	↑	TR44916_c0_g1_i1
	Glycerophosphodiester phosphodiesterase protein kinase domain−containing GDPDL2	↓	↑	↓	TR46484_c0_g1_i2
	Putative 1−phosphatidylinositol−3−phosphate 5−kinase FAB1D	↑	↑	↓	TR34194_c0_g1_i2
**Genetic information processing**					
	CCR4-associated factor 1 homolog	↓	↑	•	TR43774_c0_g1_i1
	DNA helicase	↑	↑	↓	TR3254_c0_g1_i1
	Putative transcription elongation factor SPT5	↑	↓	↓	TR33461_c0_g1_i1
	Protein translation factor SUI1 homolog	↓	↑	↑	TR2718_c0_g1_i1
	Serine/arginine−rich splicing factor RS40	↑	↓	↑	TR3607_c0_g1_i2
	Eukaryotic translation initiation factor 3 subunit A	↑	↓	↓	TR22446_c0_g1_i3
	CLK4−associating serine/arginine rich protein	↑	↑	↓	TR31855_c0_g2_i1
	Far upstream element−binding protein 3	↑	↓	↓	TR1721_c0_g1_i1
	Regulator of rDNA transcription protein 15	↓	↑	↓	TR44904_c0_g2_i1<br>TR8384_c0_g1_i1
	Heterogeneous nuclear ribonucleoprotein 1	↑	•	↓	TR36557_c0_g1_i1
	Histone H3.3	↑	↑	↓	TR28838_c2_g1_i1
	FIP1[V]−like protein	↑	↑	↓	TR26809_c1_g2_i2

↑ Up-regulated, ↓ Down-regulated, • Fold-change ≥ -1 and ≤ 1

Table S4. Table providing the link and corresponding reference for the tools and databases used in this study

Tool or database	Link	Reference (if available)
FastQC	https://www.bioinformatics.babraham.ac.uk/projects/fastqc/	-
Trimmomatic	http://www.usadellab.org/cms/?page=trimmomatic	[1]
Trinity	https://github.com/trinityrnaseq/trinityrnaseq/wiki	[2]
BUSCO	https://busco.ezlab.org/	[3]
SwissProt	www.uniprot.org	[4]
TrEMBL	www.uniprot.org	[4]
GO	http://geneontology.org/	[5]
eggNOG	http://eggnogdb.embl.de/#/app/home	[6]
KEGG	https://www.kegg.jp/kegg/	[7]
Pfam	https://pfam.xfam.org/	[8]
ExPASy-Enzyme	https://enzyme.expasy.org/	[9]
MISA	https://webblast.ipk-gatersleben.de/misa/	[10]
PlantTFDB	http://planttfdb.cbi.pku.edu.cn/	[11]
KOBAS	http://kobas.cbi.pku.edu.cn/	[12]
Function Annotator	http://fa.cgu.edu.tw/	[13]
REVIGO	http://revigo.irb.hr/	[14]

References

1. Bolger, A. M., Lohse, M., & Usadel, B. (2014). Trimmomatic: a flexible trimmer for Illumina sequence data. *Bioinformatics*, **30**, 2114-2120.
2. Haas, B. J., Papanicolaou, A., Yassour, M., Grabherr, M., Blood, P. D., Bowden, J., Couger, M. B., Eccles, D., Li, B., Lieber, M. & MacManes, M. D. (2013). *De novo* transcript sequence reconstruction from RNA-seq using the Trinity platform for reference generation and analysis. *Nature Protocols*, **8**, 1494.
3. Seppey, M., Manni, M. and Zdobnov, E. M. BUSCO: Assessing genome assembly and annotation completeness in *Gene Prediction. Methods in Molecular Biology* (ed. Kollmar, M.) 227-245 (Humana, 2019).
4. Boeckmann, B., Bairoch, A., Apweiler, R., Blatter, M. C., Estreicher, A., Gasteiger, E., Martin, M. J., Michoud, K., O'Donovan, C., Phan, I. & Pilbout, S. (2003) The SWISS-PROT protein knowledgebase and its supplement TrEMBL in 2003. *Nucleic Acids Research*, **31**, 365-70.
5. Ashburner, M., Ball, C. A., Blake, J. A., Botstein, D., Butler, H., Cherry, J. M., Davis, A. P., Dolinski, K., Dwight, S. S., Eppig, J. T. & Harris, M. A. (2000). Gene ontology: tool for the unification of biology. *Nature Genetics*, **25**, 25.
6. Huerta-Cepas, J., Szklarczyk, D., Forslund, K., Cook, H., Heller, D., Walter, M. C., Rattei, T., Mende, D. R., Sunagawa, S., Kuhn, M. & Jensen, L. J. (2015). eggNOG 4.5: a hierarchical orthology framework with improved functional annotations for eukaryotic, prokaryotic and viral sequences. *Nucleic Acids Research*, **44**, 86-93.
7. Kanehisa, M. & Goto, S. (2000) KEGG: kyoto encyclopedia of genes and genomes. *Nucleic Acids Research*, **28**, 27-30.
8. El-Gebali, S., Mistry, J., Bateman, A., Eddy, S. R., Luciani, A., Potter, S.C., Qureshi, M., Richardson, L. J., Salazar, G. A., Smart, A. & Sonnhammer, E. L. (2019). The Pfam protein families database in 2019. *Nucleic Acids Research*, **47**, 427-432.
9. Bairoch, A. (2000). The ENZYME database in 2000. *Nucleic Acids Research*, **28**, 304-305.
10. Beier, S., Thiel, T., Münch, T., Scholz, U., & Mascher, M. (2017). MISA-web: a web server for microsatellite prediction. *Bioinformatics*, **33**, 2583-2585.
11. Jin, J., Tian, F., Yang, D. C., Meng, Y. Q., Kong, L., Luo, J., & Gao, G. (2016). PlantTFDB 4.0: toward a central hub for transcription factors and regulatory interactions in plants. *Nucleic Acids Research*, **45**, 1040-1045.

12. Xie, C., Mao, X., Huang, J., Ding, Y., Wu, J., Dong, S., Kong, L., Gao, G., Li, C. Y. & Wei, L. (2011). KOBAS 2.0: a web server for annotation and identification of enriched pathways and diseases. *Nucleic Acids Research*, **39**, 16-22.
13. Chen, T. W., Gan, R. C., Fang, Y. K., Chien, K. Y., Liao, W. C., Chen, C. C., Wu, T. H., Chang, I. Y., Yang, C., Huang, P. J. & Yeh, Y. M. (2017). FunctionAnnotator, a versatile and efficient web tool for non-model organism annotation. *Scientific Reports*, **7**,10430.
14. Supek, F., Bošnjak, M., Škunca, N., & Šmuc, T. (2011). REVIGO summarizes and visualizes long lists of gene ontology terms. *PloS One*, **6**, e21800.
